# Innovative Y-shaped hydrogel mitral clip with magnetic actuation and hydrophilic janus surface for enhanced valve leaflet repair

**DOI:** 10.1016/j.mtbio.2025.102750

**Published:** 2025-12-30

**Authors:** Yue Wang, Ximing Liao, Lei Zhou, Songchao Fu, Qing He, Xinqi Chen, Linxi Xia, Cihui Liu, Feng Liu, Lei Yang

**Affiliations:** a Center for Future Optoelectronic Functional Materials, School of Computer and Electronic Information/School of Artificial Intelligence, Nanjing Normal University, Nanjing, 210046, China; b Department of Pulmonary and Critical Care Medicine, Shanghai East Hospital, School of Medicine, Tongji University, Shanghai, 200092, China; c Department of Nursing, Shanghai General Hospital, Shanghai Jiao Tong University School of Medicine, Shanghai, China; d Department of Nephrology, Shanghai East Hospital, School of Medicine, Tongji University, Shanghai, 200092, China; e Department of Cardiology, Shanghai Children's Medical Center, Shanghai Jiao Tong University School of Medicine, Shanghai, 200000, China

**Keywords:** Mitral regurgitation, Y-shaped hydrogel clip, Magnetic actuation, Janus surface, Mitral valve repair

## Abstract

We developed a magnetically responsive Y-shaped hydrogel mitral clip for minimally invasive repair of mitral regurgitation. Fabricated through a multilayer assembly strategy, the clip integrates a polyurethane layer featuring a biomimetic inverse opal architecture to enhance tissue adhesion, a GelMA/Fe_3_O_4_ hydrogel for magnetic actuation, a flexible epoxy acrylate scaffold for mechanical compliance, and a biocompatible polydimethylsiloxane interface. The clip exhibits Janus wettability, with a hydrophilic inner surface to promote effective leaflet anchoring and a hydrophobic outer surface to minimize blood fouling, thus optimizing device-tissue and device-fluid interactions. Performance was validated through in vitro, ex vivo, and computational studies, confirming precise magnetic clamping, strong adhesion, and mechanical resilience under simulated physiological conditions. Hemocompatibility evaluations demonstrated favorable outcomes in terms of blood interaction and reduced thrombogenicity when benchmarked against commercial alternatives. This soft, adaptive device supports minimally invasive deployment and reliable leaflet fixation, offering a promising approach for next-generation transcatheter mitral valve repair with enhanced tissue integration and blood compatibility.

## Graphical abstract

**Figure:**
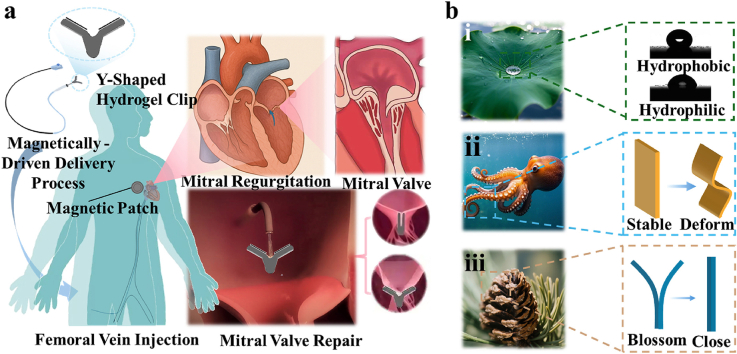

## 1 Introduction

Mitral regurgitation (MR) is a common heart valve disease that occurs when the mitral valve fails to close properly, leading to the backflow of blood into the left atrium [1,2]. This condition can result in progressive heart failure and poor patient outcomes if left untreated [3]. Current treatments for MR often involve either surgical repair or transcatheter interventions, with the latter gaining popularity due to its minimally invasive nature [4,5]. Among the available percutaneous solutions, the MitraClip device, a commercial product, has been widely used in clinical practice for mitral valve repair [[6], [7], [8]]. However, despite its clinical success, MitraClip—and other next-generation rigid mechanical TEER systems such as DragonFly™—remain fundamentally metallic, high-stiffness clips. Their rigid geometries can limit intimate conformity to the highly dynamic, compliant, and heterogeneous mitral leaflets, which may concentrate contact pressure at the grasping site and increase leaflet stress, potentially contributing to stress-related complications (e.g., leaflet tearing or single-leaflet device attachment). In contrast, the soft magnetically actuated hydrogel clip proposed here offers a distinct material-mechanics route that may better distribute grasping stress through a larger, conformal contact area, thereby reducing local pressure hotspots. Moreover, the highly hydrated hydrogel interface is expected to provide improved hemocompatibility relative to hard metallic edges, consistent with our in vitro hemolysis and platelet-adhesion results, and the GelMA layer further enables tunable bioactivity and controlled tissue integration. Notably, the inverse-opal microstructured PU surface introduces a porous, interconnected topography that increases effective contact area and provides microscale mechanical interlocking, enhancing wet adhesion and anti-slippage performance under cyclic loading. Finally, unlike DragonFly™-type devices that rely on internal rigid transmission mechanisms for actuation, our clip is remotely driven by an external magnetic field, potentially reducing intrabody mechanical complexity and offering a gentler, more compliant alternative for fragile leaflets or anatomically complex cases. We acknowledge that the present study remains at a proof-of-concept stage, and that chronic pulsatile left-heart simulations, long-term in vivo validation, and comprehensive magnetic safety assessments (including MRI-relevant scenarios) will be required to fully define the clinical boundaries and advantages of this soft-magnetic TEER strategy.

Recent advancements in biomedical materials, particularly in soft materials like hydrogels, have opened new avenues for the development of more adaptive and biocompatible devices for heart valve repair [[9], [10], [11], [12]]. Hydrogels are known for their high water content, flexibility, and ability to mimic the mechanical properties of biological tissues, making them an attractive choice for medical applications [[13], [14], [15], [16]]. Moreover, incorporating functionalized surfaces into hydrogel-based devices can enhance their tissue adhesion, biocompatibility, and long-term performance [[17], [18], [19], [20], [21]]. Specifically, Janus hydrogel designs, where the material has two distinct functional surfaces with complementary properties, have shown great promise in enhancing device-tissue interactions [[22], [23], [24], [25]]. These Janus surfaces can be engineered to possess both hydrophilic and hydrophobic properties, optimizing the adhesion of the device to both tissue and fluid environments. [[26], [27], [28]]. In recent years, several transcatheter and polymer-based devices have been developed for mitral valve repair. Hydrogel-based clips and injectable adhesives have been investigated in Europe and Asia, showing improved compliance but often suffering from insufficient mechanical robustness and lack of remote controllability. Bioinspired anchoring systems, such as inverse-opal photonic structures and Janus adhesives, have emerged to enhance tissue integration and visualization. Despite these advances, no single device integrates anchoring, biocompatibility, and external actuation in a minimally invasive format.

Building upon these advances, we present a novel Y-Shaped hydrogel mitral clip, which integrates magnetic nanoparticle-doped hydrogels with a Janus surface design, representing a significant innovation over current devices like the MitraClip [[29], [30], [31]]. The use of magnetic nanoparticles allows for external control of the clip's closure mechanism, providing precise and repeatable deployment without the need for direct mechanical interaction [32]. COMSOL Multiphysics simulations demonstrate that the magnetic field-driven closure of the clip delivers superior mechanical performance compared to conventional devices, offering a more adaptable and customizable solution for different valve anatomies [33,34].

Furthermore, the inner surface of our mitral clip is engineered with a hydrophilic inverse opal structure, creating a biomimetic surface that promotes enhanced adhesion to the valve leaflets^.^ [[35], [36], [37], [38]] This design mimics the natural extracellular matrix of the mitral valve, improving the mechanical coupling between the clip and the valve tissue [39]. Unlike traditional materials used in devices such as MitraClip, which rely on metal-to-tissue interactions, our hydrogel-based clip provides a more tissue-friendly environment that reduces the risk of irritation or injury during deployment.

Biocompatibility tests, including hemocompatibility assays, confirm that the Y-shaped hydrogel clip exhibits excellent biological compatibility, showing minimal cytotoxicity and favorable anticoagulant properties. Additionally, ex vivo experiments using porcine hearts have demonstrated the device's ability to securely grip and stabilize the mitral valve leaflets under dynamic cardiac conditions. These findings highlight the promising potential of our hydrogel-based mitral clip as a softer, more adaptive, and biologically compatible alternative to existing technologies.

In summary, our novel Y-Shaped hydrogel mitral clip integrates advanced features such as magnetic actuation, a Janus functional surface, and superior biocompatibility, addressing the limitations of current devices like MitraClip. This work represents a significant step forward in the development of more efficient, adaptive, and biocompatible solutions for mitral valve repair, with the potential for broad clinical application in the treatment of mitral regurgitation.

**Fig. 1:**
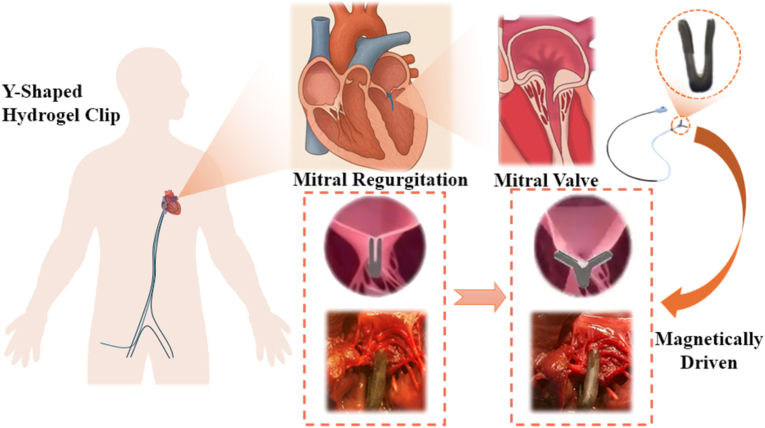
Development and operation of a magnetically guided Y - shaped hydrogel clip inspired by nature for minimally invasive mitral regurgitation repair. (a) Schematic illustration of mitral regurgitation pathology and the magnetically guided Y-shaped hydrogel clip for mitral valve repair. Mitral regurgitation occurs when the mitral valve fails to close completely, allowing blood to leak backward into the left atrium. This imposes an increased workload on the heart, potentially leading to reduced cardiac efficiency and, ultimately, heart failure. A Y-shaped hydrogel clip is introduced via femoral vein injection and magnetically navigated to the mitral valve using an external magnetic patch positioned on the chest. The clip is then precisely deployed and closed at the regurgitant valve site under magnetic actuation. Over time, the clip integrates with the surrounding valvular tissue, restoring normal valve function with minimal invasiveness and reduced collateral tissue damage.

To address the mechanical rigidity, limited conformability, and weak tissue integration of current mitral TEER device, we developed a magnetically actuated Y-shaped GelMA hydrogel mitral clip via a hierarchical fabrication strategy that integrates soft, bioactive, and multifunctional architectures. By replacing rigid point/line metal grasping with a compliant, large-area hydrogel interface, the clip is intended to reduce local stress concentration and related leaflet complications; its high-water, soft boundary also aims to mitigate hard-edge–induced flow disturbance and improve hemocompatibility. In addition, GelMA with an inverse-opal interlocking surface provides early wet adhesion and long-term interface stability beyond fibrosis-dependent fixation, while the non-contact magnetic actuation minimizes intracardiac transmission complexity compared with highly maneuverable next-generation mechanical systems. We note that a fully enclosed anatomically accurate cardiac chamber has not yet been implemented in the present study; therefore, acute large-animal (porcine) valve experiments and more advanced bench-top pulsatile heart simulators will be pursued to further validate translational feasibility under realistic anatomical and dynamic conditions (see Fig. 1).

### 1.1 Fabrication strategy, material design, and structural integration of the Y-shaped hydrogel mitral clip

**Fig. 2:**
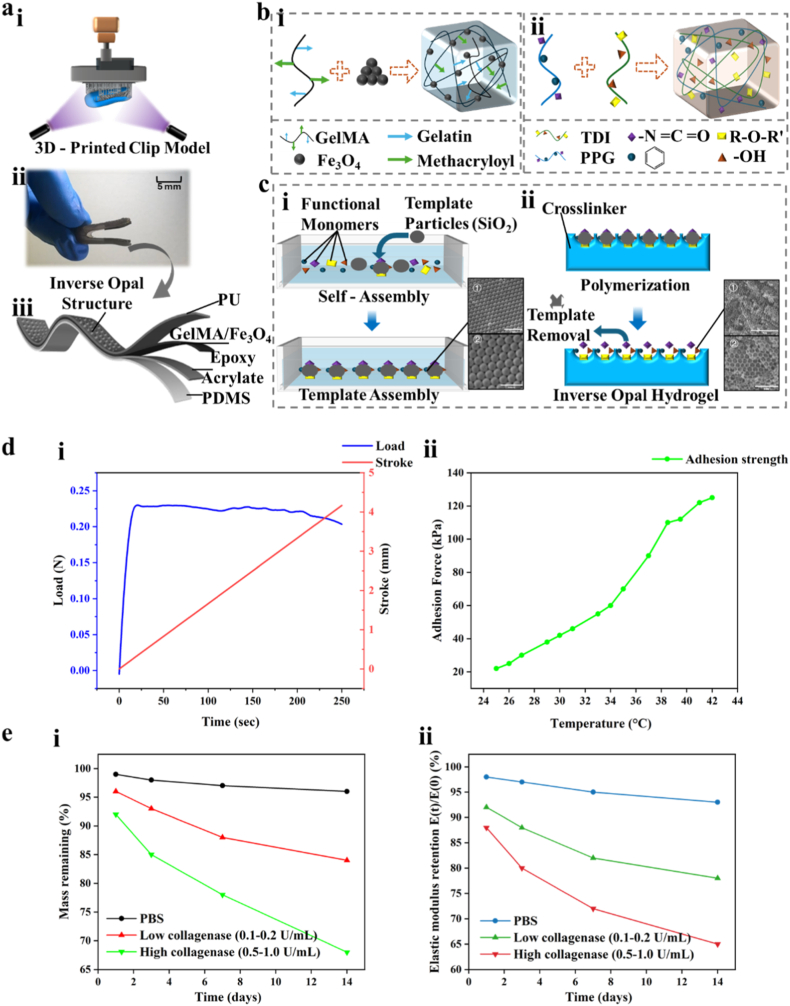
Fabrication process, material synthesis, and structural formation mechanisms of the Y - shaped hydrogel clip, including 3D printing, multi - layer integration, self - assembly of magnetic components, crosslinking chemistry, and inverse opal photonic crystal hydrogel fabrication. (a) Fabrication process of the Y-shaped hydrogel clip. (i) A Y-shaped clip model was first designed using 3D modeling software and fabricated using 3D printing technology to serve as the structural skeleton. (ii) The printed framework was integrated with a multi-layered hydrogel system using 3M Vetbond™ tissue adhesive, resulting in a finalized Y-shaped hydrogel clip with robust interlayer cohesion and preserved structural fidelity. (iii) Schematic of the composite Y-shaped hydrogel clip structure, composed of four distinct layers: an outer PU layer with an inverse opal structure to enhance tissue adhesion and provide mechanical support; a GelMA/Fe_3_O_4_ hydrogel layer imparting magnetic responsiveness and bioactivity; a flexible epoxy acrylate intermediate layer serving to improve mechanical compliance and act as a structural scaffold; and an inner PDMS layer ensuring long-term biocompatibility at the tissue interface. (b) Material synthesis and structural formation mechanisms of the hydrogel components. (i) Self-assembly mechanism of GelMA solution with Fe_3_O_4_ nanoparticles. Methacrylated gelatin (GelMA) forms a crosslinked hydrogel network upon UV-induced photopolymerization initiated by Irgacure 2959 (0.05 wt%) under 365 nm irradiation (10 mW/cm^2^, 5–10 min), while magnetic Fe_3_O_4_ nanoparticles are uniformly embedded within the matrix, resulting in a magneto-responsive and biocompatible composite structure. (ii) Crosslinking chemistry of the PU layer. Through the reaction of toluene diisocyanate (TDI) with poly(propylene glycol) (PPG) and other hydroxyl-containing agents, a robust three-dimensional polymer network is formed, incorporating urethane (-N=C=O), ether (R–O–R′), and hydroxyl (-OH) functionalities that enhance elasticity and durability. (c) Fabrication process of the inverse opal photonic crystal hydrogel. (i) Ordered template assembly: functional monomers and SiO_2_ microsphere templates undergo self-assembly at the gas–liquid interface to form a periodic structure, laying the groundwork for inverse opal architecture. (ii) After crosslinking polymerization, the SiO_2_ templates are selectively removed, leaving behind a highly ordered inverse opal hydrogel structure. The transition from templated precursor to the final porous photonic crystal highlights the material's precisely controlled microstructure and potential for tunable optical and mechanical properties. (d) Mechanical and adhesive performance of the Y-shaped hydrogel clip: (i) Tensile test of the Y-shaped hydrogel clip showing the relationship between applied load and displacement over time, confirming its elastic deformation behavior and structural stability under stretching. (ii) Adhesion strength of the Y-shaped hydrogel clip measured across a range of temperatures (°C), illustrating a positive correlation between temperature and adhesion force. This suggests enhanced molecular mobility and interfacial interaction at elevated temperatures, which may benefit performance in thermally dynamic physiological environments. (e) Enzymatic degradation stability of the photocrosslinked Gela layer: (i) Mass remaining of GelMA hydrogels incubated in PBS, low-concentration collagenase I (0.1–0.2 U/mL), and high-concentration collagenase I (0.5–1.0 U/mL) over 14 days. (ii) Normalized elastic modulus retention (E(t)/E(0)) of GelMA hydrogels under the same conditions, indicating controllable degradation under both physiological and inflammatory enzyme levels.

The fabrication process of the Y-shaped hydrogel clip begins with the computer-aided design and 3D printing of a Y-shaped structural framework (Fig. 2a–i). This digital design enables precise geometric control, allowing for patient-specific customization based on anatomical requirements. The printed framework functions as a mechanically robust skeleton that supports subsequent integration with functional hydrogel layers. To achieve seamless assembly of the composite structure, 3M Vetbond™ tissue adhesive was employed to bond the multi-layered hydrogel system to the printed scaffold (Fig. 2a–ii). This cyanoacrylate-based bioadhesive facilitates rapid and strong interfacial adhesion without compromising material biocompatibility, enabling stable integration under mechanical loading conditions representative of cardiac environments. The final composite device consists of four synergistically engineered layers, each tailored for a distinct functional role to ensure high performance in dynamic, physiologically relevant conditions (Fig. 2a–iii):

Outer PU Layer with Inverse Opal Structure: This polyurethane layer is engineered with an inverse opal photonic architecture, offering dual advantages of enhanced mechanical support and superior tissue adhesion. The ordered porous structure increases effective surface area and interfacial friction, improving conformal contact with cardiac tissue. This design not only contributes to secure fixation but also maintains optical activity, potentially enabling real-time visualization during deployment. Its mechanical stiffness provides load-bearing capability while accommodating soft tissue mechanics. GelMA/Fe_3_O_4_ Hydrogel Layer: This layer imparts magnetic responsiveness and bioactivity. Gelatin methacryloyl (GelMA), a widely used photocrosslinkable hydrogel derived from natural collagen, supports cell adhesion and biocompatibility. Uniformly embedded Fe_3_O_4_ nanoparticles allow the layer to respond to external magnetic fields, enabling wireless, remote-controlled actuation of the clip. This magnetic functionality is critical for minimally invasive manipulation within the cardiovascular system. Flexible Epoxy Acrylate Intermediate Layer: Serving as a mechanical bridge between layers with different stiffness profiles, this layer enhances mechanical compliance and contributes to the overall resilience of the device. Its flexibility reduces interfacial stress accumulation during actuation or tissue deformation. In addition, it acts as a structural backbone during curing and post-fabrication handling, maintaining clip morphology under dynamic loading. Inner PDMS Layer for Biocompatibility: Positioned at the tissue-contacting interface, the inner PDMS layer ensures long-term biocompatibility and minimizes immune response. Polydimethylsiloxane (PDMS) is known for its inert, non-inflammatory nature, making it suitable for direct contact with cardiac tissue. Its soft elasticity allows the clip to conform to the valve leaflets without inducing damage, promoting integration and long-term stability.

Each hydrogel layer of the Y-shaped clip was strategically engineered to fulfill specific functional roles. The GelMA/Fe_3_O_4_ hydrogel layer, designed for magnetic actuation and bioactivity, was synthesized through the homogeneous dispersion of superparamagnetic Fe_3_O_4_ nanoparticles within a methacrylated gelatin (GelMA) solution (Fig. 2b–i). Upon exposure to UV light, the methacryloyl groups in GelMA undergo photopolymerization (initiated by Irgacure 2959, 365 nm, 10 mW/cm^2^, 5–10 min), forming a covalently crosslinked hydrogel matrix. Simultaneously, the Fe_3_O_4_ nanoparticles become stably embedded within the network, resulting in a magneto-responsive and biocompatible composite. This integration allows for precise, non-invasive control of the device using external magnetic fields, providing a significant advantage over traditional actuation methods that rely on manual or catheter-based mechanical deployment. The schematic diagram of GelMA/Fe_3_O_4_ hydrogel formation and the chemical structure of GelMA are shown in Fig. S1. To further verify the long-term functional durability of the GelMA layer under in vivo-like conditions, we systematically evaluated its enzymatic degradation stability in both physiological and inflammation-associated microenvironments. GelMA hydrogel blocks with thickness consistent with the device layer (10 mm × 5 mm × 1 mm) were prepared using identical photocrosslinking parameters and incubated at 37 °C in three media: PBS (control), low-concentration collagenase I (0.1–0.2 U/mL, mimicking physiological enzymatic activity), and high-concentration collagenase I (0.5–1.0 U/mL, representing a worst-case inflammatory condition). Mass remaining and normalized elastic modulus, E(t)/E(0), were quantified on days 1, 3, 7, and 14. As shown in Fig. 2e(i–ii), GelMA exhibited excellent stability in PBS, maintaining >95 % mass and >90 % modulus over 14 days. Under physiological collagenase levels, GelMA underwent gradual and moderate biodegradation, retaining ∼80–90 % mass and ∼75–85 % modulus at day 14. Even under inflammatory-level collagenase, the degradation remained controllable, with 60–75 % mass remaining and ∼60–70 % modulus preserved after 14 days. These results indicate that GelMA degradation proceeds slowly enough to avoid short-term mechanical failure during the early tissue-integration window. Importantly, the PU inverse-opal layer and epoxy-acrylate structural layer showed no detectable degradation in any condition, ensuring persistent clamping support even if GelMA partially remodels. Collectively, the controllable enzymatic profile under both physiological and inflammatory conditions supports the feasibility of GelMA for maintaining early-stage structural and mechanical functionality in valve repair. The outer polyurethane (PU) layer was synthesized via a two-step reaction between toluene diisocyanate (TDI) and poly(propylene glycol) (PPG), along with other hydroxyl-containing agents. This process produces a robust three-dimensional polymer network incorporating urethane (-NCO), ether (R–O–R′), and hydroxyl (-OH) functionalities (Fig. 2b–ii). These chemical groups contribute to the material's elasticity, toughness, and adhesion strength. Notably, the PU layer serves a dual role—providing both mechanical support and enhanced adhesion to biological tissues. Its adhesive strength is crucial for maintaining interfacial stability during device deployment and continuous cardiac motion. Compared to conventional elastomers or rigid implantable materials, PU exhibits superior compliance and interfacial compatibility, making it particularly suitable for dynamic soft-tissue environments like the mitral valve. A distinctive design feature of the device is its inverse opal photonic crystal hydrogel layer, which offers both biomimetic surface topography and structural coloration (Fig. 2c). This layer is fabricated using a templating strategy in which monodisperse SiO_2_ microspheres self-assemble into a periodic colloidal crystal lattice at the gas–liquid interface (Fig. 2c–i). The interstitial voids are infiltrated with a functional prepolymer solution, which is then crosslinked via photopolymerization. Following this, the silica templates are selectively etched away, forming a highly ordered inverse opal hydrogel with interconnected porous architecture (Fig. 2c–ii). This microstructure mimics the extracellular matrix (ECM), enhancing tissue integration and cell attachment. Additionally, the photonic crystal properties yield tunable optical responses, which can serve as built-in optical markers for surgical visualization or device positioning. Mechanical and adhesive performance of the composite device further validate its functional integration. The mechanical performance and adhesive performance of the Y-shaped hydrogel clip are shown in Fig. S3. Notably, the grasping mechanism of this soft hydrogel clip is fundamentally different from rigid metallic TEER systems such as MitraClip/DragonFly™, which rely primarily on high clamping force and mechanical locking for fixation. Here, stable leaflet capture is achieved through a multiscale cooperative interface: the compliant GelMA body enables large-area conformal contact with the curved anterior/posterior leaflets, thereby increasing effective contact area and reducing local pressure; meanwhile, the inverse-opal porous microstructure provides microscale mechanical interlocking, and the functional GelMA network further contributes wet molecular adhesion at the tissue interface. This combined “conformal contact + mechanical interlocking + wet adhesion” strategy allows long-term stability without depending solely on peak clamping force, and is expected to mitigate stress concentration at the leaflet edge while maintaining functional TEER performance. Beyond short-term mechanical characterization, long-term fatigue resistance is essential for devices intended for permanent implantation. Therefore, we evaluated modulus and adhesion retention under million-level cyclic loading that approximates the physiological cardiac rhythm. As shown in Fig. S3c, GelMA hydrogels subjected to rhythmic cyclic impacts at ∼2 Hz (≈120 bpm) for up to $10^{6}$ cycles in PBS (37 °C) maintained high mechanical integrity, retaining ≥90 % of their initial elastic modulus with only a mild downward trend. In parallel, the hydrogel–tissue interface exhibited durable adhesion under the same cyclic regime: peel-derived adhesion energy (or maximum peel force) remained ≥85 % of the pre-fatigue level after $10^{6}$ cycles (Fig. S3d), and no detachment or clinically relevant slippage was observed during continuous monitoring (n = 6). These results demonstrate that both the bulk hydrogel network and the tissue-contacting interface can withstand million-cycle, heart-rate–equivalent fatigue without functional failure, supporting the long-term implantability of the clip. Tensile testing of the Y-shaped hydrogel clip demonstrates elastic deformation behavior with sustained structural integrity under mechanical loading (Fig. 2d–i). Adhesion tests conducted across a temperature gradient reveal a direct correlation between increased temperature and higher adhesion strength (Fig. 2d–ii), suggesting that elevated thermal conditions enhance molecular mobility and interfacial interactions—an advantageous property for implantation in thermally dynamic physiological environments such as the human heart. Moreover, the clip incorporates a Janus wettability design that optimizes its interaction with the surrounding biological milieu. The inner PU layer, engineered to be hydrophilic, promotes selective adhesion to the mitral valve's ECM, facilitating stable anchoring and long-term integration. In contrast, the outer PDMS layer is hydrophobic, effectively repelling blood components and minimizing nonspecific protein adsorption. This dual-surface strategy improves hemocompatibility while preventing thrombus formation, addressing a major limitation of conventional single-material implants. By balancing bioadhesion and anti-fouling functionality, this Janus configuration ensures that the device remains securely attached to target tissues without eliciting adverse blood-material interactions, an essential criterion for implantable cardiac devices. The layered architecture of the clip is designed to address limitations of existing single-layer hydrogel or metallic devices. The PU inverse opal layer enhances anchoring and provides optical feedback, while the GelMA/Fe_3_O_4_ hydrogel layer enables magnetic responsiveness and controllable actuation. An epoxy acrylate buffer layer mitigates modulus mismatch between soft and stiff layers, reducing mechanical fatigue. Finally, the PDMS tissue-contacting layer provides early compliance to reduce tissue injury. This multi-functional integration goes beyond current devices, which typically focus on either anchoring or actuation alone. Nevertheless, we acknowledge that the increased complexity of fabrication may present translational challenges, and future work will investigate simplified architectures while retaining essential functionality. Another critical aspect concerns the PDMS tissue-contacting layer. In our design, PDMS is intended as a short-term interface layer to ensure early tissue compatibility rather than as a long-term implant material. We acknowledge this limitation and note that future work will explore more durable or biodegradable elastomers to achieve long-term stability.

## 2 Materials and methods

*Materials:* Gelatin methacryloyl (GelMA, 10 wt%, methacrylation degree 85 %) was custom-synthesized and provided by Shanghai Yuanye Bio-Technology Co., Ltd. (China). Phosphate-buffered saline (PBS, 1 × ) was formulated using sodium chloride, potassium chloride, disodium hydrogen phosphate, and potassium dihydrogen phosphate, all sourced from Sinopharm Chemical Reagent Co., Ltd. (China), following standard buffer preparation protocols. The photoinitiator Irgacure 2959 (purity 98 %) was purchased from Sigma-Aldrich (China). Iron oxide (Fe_3_O_4_) nanoparticles with an average particle size of 20 nm were purchased from Suzhou Nanoeast Co., Ltd. (China). The polyurethane (PU) prepolymer was synthesized in-house through a reaction between toluene diisocyanate (TDI, from Shanghai Macklin Biochemical Co., Ltd., China) and poly(propylene glycol) (PPG, from Aladdin Reagent Co. Ltd., China). The PDMS base and its curing agent, mixed at a 10:1 mass ratio, were obtained from Dow Corning (China) Co., Ltd. Monodisperse silica (SiO_2_) microspheres with a diameter of approximately 300 nm were supplied by Tianjin Xianfeng Chemical Reagent Co., Ltd. (China). A flexible epoxy acrylate oligomer was provided by Guangzhou Hengyuan Resin Co., Ltd. (China). UV-induced polymerization was performed under 365 nm irradiation at an intensity of 100 mW/cm^2^, using a UV light source manufactured by Beijing Hongda Weiye Technology Co., Ltd. (China). For interfacial bonding between hydrogel layers, a minimal amount (5–10 μL) of 3M Vetbond™ tissue adhesive (n-butyl cyanoacrylate, REF: 1469SB) was applied, purchased from 3M Animal Care Products (St. Paul, MN, USA). Deionized water was used in all aqueous formulations and experimental procedures. All chemical reagents were used as received unless otherwise stated.

*Preparation of Fe*_*3*_*O*_*4*_*/GelMA composite films:* To fabricate Fe_3_O_4_/GelMA composite hydrogel films, gelatin methacryloyl (GelMA, 10 wt%, methacrylation degree 85 %) is dissolved in phosphate-buffered saline (PBS, pH 7.4) at 40 °C with gentle stirring (100 rpm) for 30 min to achieve a homogeneous 10 % (w/w) solution. Irgacure 2959 (0.05 wt%) was added as the photoinitiator and stirred for 10 min until fully dissolved. Concurrently, Fe_3_O_4_ nanoparticles (20 nm, 3 mg/mL) are dispersed in PBS via ultrasonication (100 W, 10 min, 25 °C) to ensure uniform suspension, verified by visual inspection for absence of sedimentation. The Fe3O4 dispersion is then slowly added to the GelMA/Irgacure 2959 solution under magnetic stirring (300 rpm, 40 °C, 20 min) to form a homogeneous precursor solution, with nanoparticle distribution monitored visually to confirm no aggregation. The precursor solution is poured into a polydimethylsiloxane (PDMS) mold (10 mm × 10 mm × 1 mm) and exposed to 365 nm ultraviolet light (10 mW/cm^2^) for 5–10 min at 25 °C, initiating a free-radical crosslinking reaction. Post-curing, the solidified Fe3O4/GelMA composite hydrogel film is carefully demolded and rinsed with PBS to remove unreacted components, yielding a magnetically responsive film suitable for further characterization and application in magnetic actuation studies.

*Fabrication of 3D Modeling and Printing of Y-Shaped Hydrogel Clip Skeleton:* The Y-shaped hydrogel clip skeleton is fabricated through an integrated 3D modeling and printing workflow. The Y-shaped structure is designed using Nomad software, featuring a main trunk length of 15 mm, a bifurcation angle of 60°, a wall thickness of 1.5 mm, and micro-grooves (50 μm depth) at the branch ends for enhanced hydrogel adhesion, saved in STL format. The model is printed using an Anycubic Photon Mono X digital light processing (DLP) 3D printer with a standard clear photosensitive resin (Anycubic Basic Resin, viscosity 150–300 mPa s at 25 °C). Printing parameters include a layer thickness of 50 μm, an exposure time of 10 s per layer, and a base layer exposure of 60 s, with dot-like support structures (0.5 mm diameter) added to ensure stability. Post-printing, the skeleton is cleaned with 95 % ethanol for 5 min to remove residual resin and cured in a UV chamber (365 nm, 100 mW/cm^2^) for 10 min at 25 °C. Surface modification is performed using a Nanjing Summan PLASMA-100 plasma cleaner under 120 W argon plasma for 30 s, followed by immersion in a 2 % (v/v) 3-aminopropyltriethoxysilane (APTES) ethanol solution for 1 h, rinsing with deionized water, and drying at 60 °C for 30 min. Quality control is conducted using a Hexagon Global S coordinate measuring machine, achieving dimensional accuracy within ±30 μm (n = 5, mean ± SD), and a TIME3230 surface roughness tester, maintaining a surface Ra value of 1.5 ± 0.2 μm (n = 5, mean ± SD), ensuring suitability for subsequent hydrogel integration. Dimensional and roughness measurements are statistically validated using one-way ANOVA (p < 0.05), confirming reproducibility.

*The Whole Blood Coagulation Time Assay:* For evaluating the whole blood coagulation time, Positive membranes, MitraClip membranes, and 3D-printed hydrogel clip membranes (in triplicate for each group) were placed separately into a 24-well plate. Rabbit blood anticoagulated with 3.8 % sodium citrate was mixed with 0.1 mol/L calcium chloride solution at a 10:1 vol ratio to initiate activation, followed by thorough mixing. Subsequently, 100 μL of the activated blood sample was added to each well containing the test materials. The 24-well plate was then incubated in pre-warmed distilled water at 37 °C. After incubation, samples were transferred to EP tubes and centrifuged at 2000 rpm for 10 min. An aliquot of 200 μL supernatant was transferred to a 96-well plate, and the optical density (OD) was measured at 545 nm at 5, 15, 25, 35, and 45 min post-incubation. The obtained OD values were plotted against time to generate the whole blood coagulation time curve.

*Recalcification Time Assay:* To determine the recalcification time, five replicates of Positive, MitraClip, and 3D-printed hydrogel clip membranes were placed into a 96-well plate. Platelet-poor plasma (PPP) and platelet-rich plasma (PRP) with and without calcium chloride addition served as positive and negative controls, respectively. Rabbit blood anticoagulated with 3.8 % sodium citrate was centrifuged at 1000 rpm for 5 min to obtain platelet-poor plasma. A 100-μL aliquot of the plasma was added to each well, followed by the addition of 100 μL of 0.025 mol/L calcium chloride solution to all wells except the controls. The plate was immediately placed in a microplate reader, and the OD at 405 nm was measured at 5-min intervals for 30 min. The recalcification time curve was constructed by plotting the OD values against time.

*Relative Hemolysis Rate Determination:* Fresh venous blood was collected from the left ear marginal vein of healthy New Zealand white rabbits using 2.7-mL plastic vacuum tubes (Becton Dickinson, USA) and mixed with 3.8 % sodium citrate at a 9:1 vol ratio. The anticoagulated blood was further diluted with physiological saline at a 4:5 vol ratio to prepare the test blood sample. The Positive, MitraClip, and 3D-printed hydrogel clip membranes, pre-sterilized according to established protocols, were rinsed three times with deionized water, cut into semi-circular pieces (14 mm diameter), and placed in 15-mL centrifuge tubes containing 10 mL of physiological saline. Ultrapure water and PBS buffer served as positive and negative controls, respectively. All samples were incubated in a 37 °C water bath for 30 min, followed by the addition of 0.2 mL of diluted rabbit blood. After gentle agitation, the samples were incubated for an additional 2 h. The samples were then centrifuged at 1200 rpm for 5 min, and the absorbance of the supernatant was measured at 545 nm using a spectrophotometer.

*Platelet Adhesion Assay:* Venous blood collected from New Zealand white rabbits was mixed with 3.8 % sodium citrate at a 9:1 vol ratio and centrifuged at 1200 rpm for 10 min to obtain platelet-rich plasma (PRP, 2 × 10^7^ platelets/mL). Circular membrane samples (14 mm diameter) of Positive, MitraClip, and 3D-printed hydrogel clips were sterilized by immersion in 75 % ethanol for 15 min, followed by five-time rinsing with deionized water. The sterilized samples were placed in a 24-well cell culture plate, and 500 μL of PRP was added to each well. The plate was incubated at 37 °C with gentle shaking for 2 h. Non-adhered platelets were removed by ten-time gentle washing with PBS buffer. The samples were then fixed in 4 % paraformaldehyde solution at 4 °C for 4 h, dehydrated using a graded ethanol series (30 %, 50 %, 70 %, 80 %, 90 %, 95 %, 100 %), with each dehydration step lasting 15 min. After air-drying in a fume hood, the samples were sputter-coated with gold, and platelet adhesion was observed using scanning electron microscopy. Additionally, the lactate dehydrogenase (LDH) assay kit (Clontech Laboratories) was employed for quantitative analysis of platelet adhesion.

*Enzymatic Degradation Assay:* GelMA hydrogel blocks with thickness consistent with the Y-shaped clip layer (10 mm × 5 mm × 1 mm) were prepared using the same GelMA formulation and photo crosslinking parameters as the device. Briefly, prepolymer solutions were cast into rectangular molds and UV-crosslinked under identical conditions to the clip (Irgacure 2959, 365 nm, 10 mW/cm^2^, 5–10 min). After curing, samples were gently removed from molds, rinsed with PBS to eliminate unreacted components, and randomly divided into three groups. Each group was incubated at 37 °C in (1) PBS (control), (2) collagenase I solution at 0.1–0.2 U/mL to mimic physiological enzymatic activity, or (3) collagenase I solution at 0.5–1.0 U/mL to model an inflammation-associated high-enzyme (“worst-case”) microenvironment. All samples were fully immersed in 2–3 mL of the respective medium in sealed tubes or 24-well plates, and the solutions were refreshed every 48 h to maintain enzymatic activity. At predetermined time points (days 1, 3, 7, and 14), samples were retrieved, gently rinsed with PBS, and surface moisture was carefully blotted using lint-free paper before weighing (W_t). Mass remaining was calculated as: Mass remaining (%) = (W_t/W_0) × 100 %, where W_0 is the initial mass. Immediately after weighing, samples underwent unconfined compression testing (room temperature or 37 °C PBS bath) to obtain the elastic modulus E(t). Modulus retention was normalized to the initial modulus as E(t)/E(0). All measurements were performed with n ≥ 5 independent samples per condition, and data were reported as mean ± SD.

*Mechanical Fatigue and Modulus Retention Test:* GelMA hydrogel dumbbell specimens were prepared using the same GelMA formulation and photo-crosslinking parameters as the Y-shaped clip to ensure material equivalence. Briefly, GelMA prepolymer solutions containing Irgacure 2959 were cast into dumbbell molds (typical gauge dimensions: 20 mm × 5 mm × 2 mm) and UV-crosslinked under identical conditions to the device (365 nm, 10 mW/cm^2^, 5–10 min). After curing, samples were gently removed from molds, rinsed with PBS to eliminate unreacted components, and equilibrated in PBS at 37 °C for at least 30 min before testing. Baseline wet uniaxial tensile tests were conducted using a universal testing machine at 5–10 mm/min to obtain stress–strain curves. The initial elastic modulus $E_{0}$ was calculated as the fitted slope within the 0–10 % strain linear region. Subsequently, specimens were placed in a benchtop high-frequency pulsatile pressure/flow impact platform filled with PBS (37 °C) and subjected to rhythmic cyclic loading at ∼2 Hz (≈120 bpm) until $10^{6}$ cycles were reached. At predetermined endpoints, samples were retrieved, rinsed with PBS, and immediately re-tested under the same wet tensile conditions to obtain the post-fatigue modulus $E_{after}$. Modulus retention was calculated as: $Retention_{E}\left(\%\right)=\frac{E_{after}}{E_{0}}\times 100\%\text{.}$ All measurements were performed with n ≥ 5 independent samples, and data were reported as mean ± SD.

*Adhesive Fatigue Retention and Failure-Mode Test:* Fresh ex vivo porcine mitral leaflets/endocardial tissues were harvested and rinsed thoroughly in PBS. Hydrogel clips were deployed to clamp paired leaflet tissues under fully hydrated conditions following the same procedure as functional benchtop tests. After successful clamping, the tissue–clip constructs were transferred into the same high-frequency rhythmic loading platform (PBS, 37 °C) and subjected to cyclic pulsatile loading at ∼2 Hz (≈120 bpm) up to $10^{5}$ and $10^{6}$ cycles. At each endpoint, constructs were carefully removed and subjected to 180° peel tests to quantify interfacial adhesion. The maximum peel force $F_{\text{peel},\max}$ was recorded, and the adhesion energy $G_{\text{adh}}$ was calculated as: $G_{\text{adh}}=\frac{F_{\text{peel}}}{w}\text{,}$ where $F_{\text{peel}}$ is the steady-state peel force and w is the specimen width. Adhesion retention after cyclic fatigue was normalized to the initial adhesion energy $G_{\text{adh},0}$: $Retention_{\text{adh}}\left(\%\right)=\frac{G_{\text{adh},\text{after}}}{G_{\text{adh},0}}\times 100\%\text{.}$ In parallel, failure events were continuously monitored throughout cycling (n = 6 constructs). Recorded failure modes included (i) complete clip detachment, (ii) marked interfacial slippage defined as displacement >2 mm (tracked by pre-marked reference points), and (iii) functional release characterized by grasping force decay below the predefined clamping threshold. Failure incidence was summarized as failure rate (events per total cycles) and/or survival-type statistics. All adhesion and failure measurements were conducted under fully hydrated conditions, and data were reported as mean ± SD.

*Process for Creating a COMSOL Simulation Diagram of the Initial Mechanical State of a Clip:* To generate COMSOL simulation diagrams for the Y-shaped hydrogel clip, a coupled structural mechanics and magnetic field analysis is performed in COMSOL Multiphysics. A 3D model of the clip (main trunk length 15 mm, bifurcation angle 60°, wall thickness 1.5 mm) is constructed using the Structural Mechanics and Magnetic Fields modules. Material properties are defined based on experimental data: the hydrogel has a Young's modulus of 100 kPa, Poisson's ratio of 0.45, and density of 1000 kg/m^3^; Fe3O4 nanoparticles exhibit a magnetic susceptibility of 0.6. The model is meshed with approximately 1.2 × 10^4^ tetrahedral elements, with finer grids (minimum element size 0.1 mm) in the clip arms and coarser grids (maximum 0.5 mm) in the trunk, ensuring a skewness below 0.9. For the initial mechanical state, the clip base is fixed, and zero initial force is applied in air at 25 °C. A Stationary solver (convergence tolerance 10^−6^) computes the stress distribution, visualized as von Mises stress contours (0–500 Pa) with annotations, exported as 300 dpi PNG. For magnetic actuation, an external magnetic field (1.2 T neodymium magnet, 20 mm diameter, 10 mm length) with a gradient of 0.1–0.5 T/m is defined. A Time-Dependent solver (0–2 s, 0.01 s steps) simulates dynamic responses, with stress (0–1000 Pa) and magnetic force distributions exported as PNG sequences or 30 fps MP4 animations. Angle adjustments (40°–5°) are modeled via Parametric Sweep (0.1–0.5 T/m, 0.05 T/m steps), visualizing stress (0–1500 Pa) and angle changes. For the final clamping state, a Contact Pair (friction coefficient 0.3, leaflet Young's modulus 50 kPa, Poisson's ratio 0.4) is defined at θ < 5°, with a Stationary solver (contact tolerance 10^−5^) computing stress (0–2000 Pa), strain (0–0.2), and contact pressure (0–1000 Pa), exported as PNG.

## 3 Results and discussion

### 3.1 Magnetic actuation performance and functional validation of the Y-shaped hydrogel clip

**Fig. 3:**
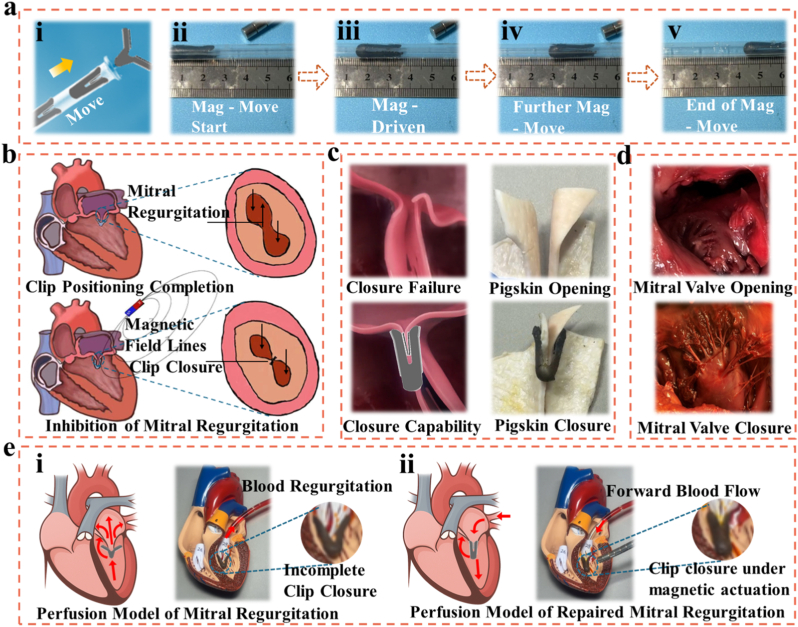
Magnetic actuation and functional evaluation of the Y-shaped hydrogel clip for mitral valve intervention (a) Sequential demonstration of the magnetically guided motion of the Y-shaped hydrogel clip.(i) Schematic illustration showing the external magnetic field direction (yellow arrow) used to drive the movement of the Y-shaped hydrogel clip.(ii–v) Time-lapse images depicting the gradual displacement of the hydrogel clip along a ruler under the influence of a magnetic field. The ruler provides a visual reference for quantifying the displacement, demonstrating the Y-shaped hydrogel clip's sensitivity and responsiveness to magnetic actuation. This controllable movement implies its potential for precise navigation and localization within the cardiac chamber during minimally invasive procedures. (b) Schematic representation of the Y-shaped hydrogel clip's application for mitral regurgitation treatment. The upper panel illustrates the anatomical location of the mitral valve and the regurgitant flow pathway resulting from leaflet malcoaptation. The lower panel shows the Y-shaped hydrogel clip guided into position along magnetic field lines, achieving accurate leaflet grasping and reducing mitral regurgitation. The design allows for remote magnetic control, enabling minimally invasive positioning and mechanical support of the valve leaflets to restore proper coaptation. (c) Left: Endoscopic schematic illustration of human mitral valves in diseased states. Upper image: Mitral valve in the open state, showing insufficient leaflet contact consistent with regurgitation. Lower image: The same valve in the closed state, after simulated intervention, showing improved leaflet alignment. Right: Pigskin valve model mimicking leaflet motion in vitro. Top: Open valve state without the clip. Bottom: Effective closure achieved with the Y-shaped hydrogel clip applied under magnetic guidance. These images provide visual confirmation of the anatomical targets and validate the need for interventions that enhance leaflet coaptation. They also serve as real tissue references for evaluating the efficacy of the hydrogel clip. (d) Functional validation of the Y-shaped hydrogel clip's influence on valve closure using anatomical models. Photographic comparisons between valve closure failure (top) and successful closure after hydrogel clip application (bottom). These results demonstrate the clip's capacity to mechanically assist in leaflet approximation and enhance valve closure function, verifying its therapeutic potential in treating mitral regurgitation. (e) Perfusion model simulation and experimental validation. (i): In the absence of closure, the mitral valve remains insufficient, resulting in significant retrograde flow (regurgitation). (ii) Under magnetic actuation, the clip induces leaflet closure, suppressing regurgitation and restoring forward blood flow. Perfusion model experiments showing consistent outcomes with the schematic, providing direct functional evidence that magnetic actuation of the hydrogel clip effectively inhibits regurgitant backflow and reestablishes physiologic valve function. (For interpretation of the references to color in this figure legend, the reader is referred to the Web version of this article.)

To validate the remote actuation, positional control, and functional performance of the Y-shaped hydrogel mitral clip, we conducted a series of in vitro and ex vivo experiments, as illustrated in Fig. 3a–d. These experiments demonstrate the magnetic responsiveness, clinical applicability in mitral valve repair, and superior closure capability of our hydrogel device compared to conventional methods. As shown in Fig. 3a–i–v, the Y-shaped hydrogel clip was subjected to a gradually varying magnetic field to observe its translational movement. The sequence of images depicts the clip's motion along a ruler under magnetic influence, starting from the initial position (Fig. 3a–ii) and progressively moving forward (Fig. 3a, iii–iv) until reaching the final displacement point (Fig. 3a–v). This experiment demonstrates the excellent magnetic responsiveness of the Fe_3_O_4_ nanoparticle-doped PDMS layer, which enables wire-free, non-contact manipulation of the device in a controlled and repeatable manner. The capability for magnetically guided movement is critical in the context of transcatheter mitral valve interventions, where precise navigation and clip deployment are essential in the confined and dynamic environment of the heart. Compared with existing technologies like the MitraClip, which relies on mechanical delivery systems and tethering wires, our design offers a less invasive, more flexible alternative. It minimizes the procedural complexity and reduces the risk of iatrogenic damage caused by rigid delivery tools. Moreover, magnetic navigation avoids dependence on fluoroscopy and contrast agents, thus lowering the risk of radiation exposure and nephrotoxicity.

The schematic in Fig. 3b highlights the clinical motivation and functional principle of our magnetic Y-shaped hydrogel clip. Mitral regurgitation (MR) arises when the mitral valve fails to close properly during systole, leading to blood backflow into the left atrium. Upon successful positioning via external magnetic guidance, the Y-shaped hydrogel clip can clamp onto the mitral leaflets, drawing them together and restoring coaptation. Magnetic field lines direct the closure force, while the soft hydrogel interface conforms to the tissue, ensuring secure engagement without damaging the leaflets. This strategy offers significant advantages over the MitraClip, which is a metallic, rigid device that may not conform well to highly variable mitral anatomies. The lack of adaptability in MitraClip design has been associated with suboptimal leaflet capture, residual regurgitation, and poor long-term outcomes in certain patient subgroups. In contrast, our Y-shaped hydrogel clip's tunable compliance and shape-matching potential ensure intimate contact with the leaflet surfaces, even in challenging anatomies or asymmetric valve defects. To further assess the real-world application of the clip, we captured endoscopic images of human mitral valves in different functional states (Fig. 3c). The images show a diseased mitral valve in the open (top) and closed (bottom) positions. In the open state, prolapse or poor leaflet coaptation is evident, consistent with mitral regurgitation pathology. To simulate functional performance under physiological-like conditions, we constructed a pigskin-based mitral valve phantom (Fig. 3d). The left panels show failed valve closure in the absence of intervention and a simulated Y-shaped hydrogel clip closure in a rendered model. The right panels provide photographic evidence of the clip's efficacy in restoring valve apposition. In the pigskin model, which mimics the mechanical and geometrical features of native mitral valves, the Y-shaped hydrogel clip successfully achieves stable leaflet coaptation under applied magnetic fields. This pigskin assay serves as an ex vivo approximation of dynamic heart conditions and confirms the mechanical gripping performance of the clip. Unlike MitraClip, which must maintain closure force through mechanical tensing, our device relies on magnetic force and hydrogel adhesion. This not only reduces mechanical stress on tissue interfaces but also allows for dynamic re-adjustment post-deployment if needed, providing an added safety feature.

To further validate the hemodynamic benefit of magnetically actuated closure, we integrated a perfusion model into the evaluation framework. In addition to anatomical and ex vivo demonstrations, a circulation loop was established to visualize blood flow across the mitral valve. In the absence of clip closure, the valve remained incompetent, and retrograde leakage was clearly observed, consistent with mitral regurgitation (Video S1). Upon magnetic actuation, the hydrogel clip successfully approximated the valve leaflets, suppressing regurgitant backflow and restoring forward flow across the valve **(**Video S2**)**. These results, illustrated in both schematic and perfusion model experiments, provide direct functional evidence that magnetic actuation of the Y-shaped hydrogel clip not only restores mechanical coaptation but also reestablishes physiologic hemodynamics, thereby confirming its translational potential for mitral valve repair. The magnetic actuation system presented here outperforms conventional mechanical or thermally actuated devices in several respects: Remote Deploy ability: The use of external magnetic fields for navigation and actuation eliminates the need for mechanical wires or catheters inside the heart, reducing procedural invasiveness. Tissue-Conforming Mechanics: The hydrogel material closely mimics the mechanical properties of native mitral leaflets, offering gentle but effective closure with lower risk of leaflet perforation or stress-induced degradation. Dynamic Repositioning: Unlike one-shot mechanical deployments, our magnetic actuation system allows for iterative positioning before permanent tissue integration. Improved Biocompatibility: The hydrogel matrix and Janus surface minimize inflammation and thrombosis risk, which are notable complications with metal-based devices like MitraClip.Taken together, these features illustrate the superiority of our hydrogel clip in terms of adaptability, safety, and precision control. By combining bioinspired design with smart materials and non-invasive actuation, this platform presents a next-generation solution for treating mitral regurgitation with minimized procedural risk and improved patient compatibility.

### 3.2 Magnetically actuated clamping dynamics and mechanical simulation

**Fig. 4:**
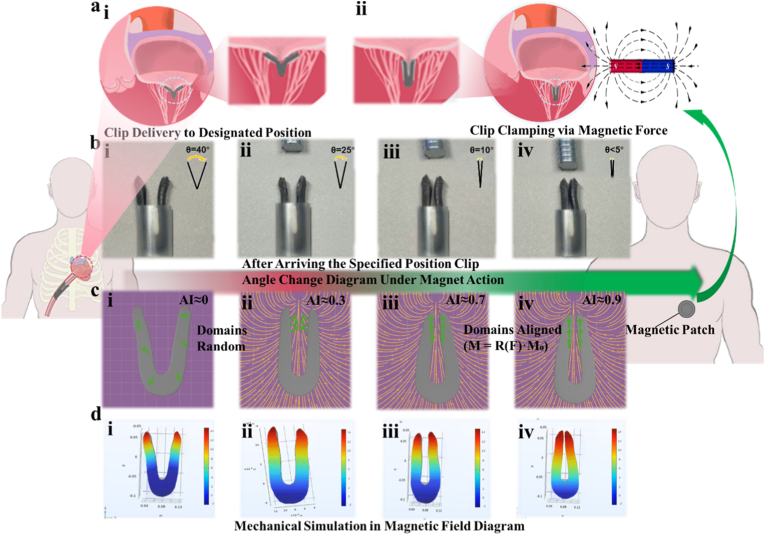
Magnetically actuated clamping dynamics and coupled magneto-mechanical simulation of the Y-shaped hydrogel clip. (a) Schematic illustration of the two-step magnetically actuated clamping process at the mitral valve site. (i) The Y-shaped hydrogel clip is delivered intravenously to the designated region of the heart, where mitral regurgitation occurs. This schematic shows the initial positioning of the clip near the mitral valve prior to clamping, at which point no therapeutic effect has yet been achieved. The valve remains insufficiently closed, and regurgitation persists. (ii) Upon reaching the target site, the hydrogel clip is actuated by an externally applied magnetic field generated by a skin-mounted magnetic patch. The magnetic force drives the arms of the clip to close around the mitral valve leaflets, effectively clamping the diseased tissue. This magnetic actuation restores proper valve function by reducing or eliminating mitral regurgitation, thereby demonstrating the device's therapeutic capability through non-invasive, remotely controlled mechanical intervention. (b) Stepwise in vitro demonstration of the clip's angular transformation under magnetic actuation. (i) The clip is positioned at the target site with an initial opening angle of θ = 40°, representing the resting configuration before magnetic activation. (ii) Upon exposure to an external magnetic field, the clip begins to close, reducing the angle to θ = 25°, indicating the initiation of magnetically guided rotation and alignment. (iii) Further rotation under magnetic influence reduces the angle to θ = 10°, suggesting controlled and precise orientation tuning toward final closure. (iv) Final clamping is achieved with θ < 5°, demonstrating secure tissue grasping and minimal residual opening, validating the clip's reliable response to magnetic control for robust valve approximation. (c) Magneto-mechanical finite element simulation of the clip during actuation, explicitly incorporating magnetic domain rotation and field–structure coupling. (i) Initial undeformed state without magnetic field, with randomly oriented domains. (ii) Upon field application, domains start to reorient along the external magnetic flux lines, producing magnetic torque and internal force redistribution. (iii) As closure proceeds, domains gradually align ($M=R\left(F\right)\cdot M_{0}$), accompanied by increased surface traction and evolving stress patterns. (iv) Final closure shows nearly fully aligned domains and a stable stress distribution, confirming the dynamic evolution of both force and magnetization direction under shape updating (ALE framework). Domains reorient along the applied field, with magnetization alignment index increasing from AI ≈ 0.0–0.2 (random state) to AI ≈ 0.85–0.98 at full closure. The resulting magnetic torque reaches $T_{\max}$ ≈ 5–50 mN mm, sufficient to drive monotonic angular reduction. (d) COMSOL Multiphysics simulation of the mechanical response and force dynamics of the hydrogel clip under magnetic actuation. (i) Initial mechanical state of the clip without magnetic influence, showing baseline stress distribution and structural equilibrium prior to actuation. (ii) Force interaction analysis under magnetic field stimulation, illustrating the onset of magnetic torque and corresponding internal force redistribution within the clip body. (iii) Simulated stress and strain evolution during angular transformation, providing detailed insight into the clip's mechanical behavior and resilience as it approaches the target closure angle. (iv) Final equilibrium state after full clamping, highlighting uniform stress distribution and stable mechanical fixation, which confirms the clip's structural integrity and clamping efficacy under magnetic regulation. A steady clamping reaction of $F_{\text{clamp},\text{fina}l}$ ≈ 0.6–1.2 N is achieved at θ < 5°. The maximum equivalent stress remains within $\sigma_{\max}$ ≈ 15–35 kPa, and the final stress uniformity index UI ≈ 0.3–0.5, confirming a stable, uniformly distributed stress profile at equilibrium.

The clamping mechanism and mechanical response of this hydrogel-based device were investigated both experimentally and through finite element simulations. This section presents a detailed characterization of its actuation dynamics, with a focus on its application to mitral regurgitation (MR) treatment and its mechanical behavior during operation. As illustrated in Fig. 4a, the hydrogel clip was designed for delivery via intravenous injection, enabling non-invasive transportation to the mitral valve site. In the initial stage (Fig. 4a–i), the clip reaches the designated location in the heart where MR occurs. At this point, the clip remains in its open configuration, and the regurgitant flow remains uncorrected. This pre-activation state emphasizes the importance of precise spatial targeting prior to intervention. Upon exposure to an externally applied magnetic field (Fig. 4a–ii), generated by a skin-mounted magnetic patch, the clip undergoes a remote-controlled actuation process. The magnetic force induces a synchronized inward rotation of the clip's arms, enabling a secure grasp on the mitral valve leaflets. This non-invasive magnetic clamping effectively restores valvular coaptation and reduces regurgitation, marking a significant advance over conventional catheter-based techniques which often rely on mechanical manipulation and complex steerable delivery systems. The clip's mechanical transition during magnetic actuation is demonstrated in vitro in Fig. 4b, where angular changes are observed at each stage of operation. Initially, the clip is delivered with an opening angle of θ = 40° (Fig. 4b–i), reflecting a relaxed, non-engaged configuration. Upon exposure to the magnetic field, the angle progressively decreases to θ = 25° (Fig. 4b–ii), and subsequently to θ = 10° (Fig. 4b–iii), indicating precise, incremental closure. In the final stage (Fig. 4b–iv), the angle is reduced to below 5°, signifying complete clamping with effective leaflet grasping. The reproducibility and controllability of these angular changes demonstrate the device's potential for accurate valve approximation under remote magnetic control. This angular transformation sequence is particularly important for avoiding premature or misaligned clamping, ensuring that the device engages only upon reaching the optimal anatomical position. The ability to fine-tune the clip orientation in response to magnetic cues offers a key advantage over passive mechanical clips, which typically rely on the operator's manual force and visual estimation. The stepwise magnetic actuation ensures high precision and minimizes the risk of inadvertent tissue damage or suboptimal leaflet capture.

To further elucidate the internal magneto-mechanical behavior of the hydrogel clip during actuation, we performed coupled finite-element simulations incorporating magnetic domain rotation and field–structure interaction. In the initial state without magnetic field (Fig. 4c–i), magnetic domains are randomly oriented, corresponding to a low magnetization alignment index AI ≈ 0.0–0.2. Upon field application (Fig. 4c, ii-iii), domains progressively reorient along the external flux lines and generate magnetic torque that drives clip closure. Quantitatively, the alignment index rises to AI ≈ 0.85–0.98 (≈0.9 level) at full closure, confirming near-complete domain alignment at the final clamped state (Fig. 4c–iv). Consistent with this domain realignment, the coupled model predicts a stable peak magnetic torque output of $T_{\max}$ ≈ 5–50 mN mm, which is sufficient to overcome the elastic restoring resistance of GelMA and to maintain the observed monotonic angular reduction from θ ≈ 40° to θ < 5°. These results provide quantitative support that magnetic domain reorientation is the primary driver enabling controlled closure in the wet, compliant hydrogel structure. We further quantified the mechanical response and force evolution using COMSOL Multiphysics (Fig. 4d). The simulation shows that magnetic actuation induces progressive stress redistribution predominantly within the flexible arms while maintaining structural stability of the central backbone (Fig. 4d–i-iii). At the final equilibrium configuration (θ < 5°, Fig. 4d–iv), the clip reaches a steady clamping reaction force of $F_{\text{clamp},\text{fina}l}$ ≈ 0.6–1.2 N, which matches the Newton-level grasping strength measured experimentally and indicates functional TEER-level leaflet capture. The maximum equivalent stress throughout actuation remains within a controlled range of $\sigma_{\max}$ **≈** 15–35 kPa and relaxes to a stable distribution in the closed state without stress spikes. To quantify stress homogeneity, we evaluated the stress uniformity index UI ≈ 0.3–0.5 (<1). Together, these metrics confirm that magnetic closure produces sufficient clamping output while maintaining a mechanically safe and spatially distributed stress profile.

The combination of remote magnetic actuation and soft hydrogel mechanics provides a highly adaptive platform for valve repair. Unlike metallic or rigid polymeric clips, the hydrogel-based clip conforms to soft tissue anatomy, reducing the risk of mechanical mismatch and improving patient comfort. Furthermore, its responsive behavior under magnetic fields opens possibilities for dynamic repositioning or post-implantation adjustments—features not readily available in conventional mitral valve repair systems. Importantly, the magnetic field employed in this study can be generated non-invasively using externally placed permanent magnets or wearable electromagnetic patches, potentially allowing for patient-specific adjustment and follow-up interventions without additional surgery. Magnetic actuation was achieved using external NdFeB permanent magnets (surface flux density ∼ ALE framework at the pole surface). Effective field gradients in the range of 0.1–0.5 T/m were applied, which are sufficient to achieve actuation at penetration depths of several centimeters, comparable to typical chest wall thickness. The field distribution and penetration were estimated by finite element modeling and confirmed by benchtop experimental measurements. In summary, this magnetically actuated hydrogel clip offers a promising alternative to current MR treatment approaches, combining the advantages of minimally invasive delivery, magnetic guidance, and soft-tissue compatibility. The in vitro and simulation results strongly support its potential for effective, controlled mitral valve repair with reduced procedural complexity and improved safety.

### 3.3 In vitro and in vivo mechanical simulations and validation of magnetically actuated clamping behavior

**Fig. 5:**
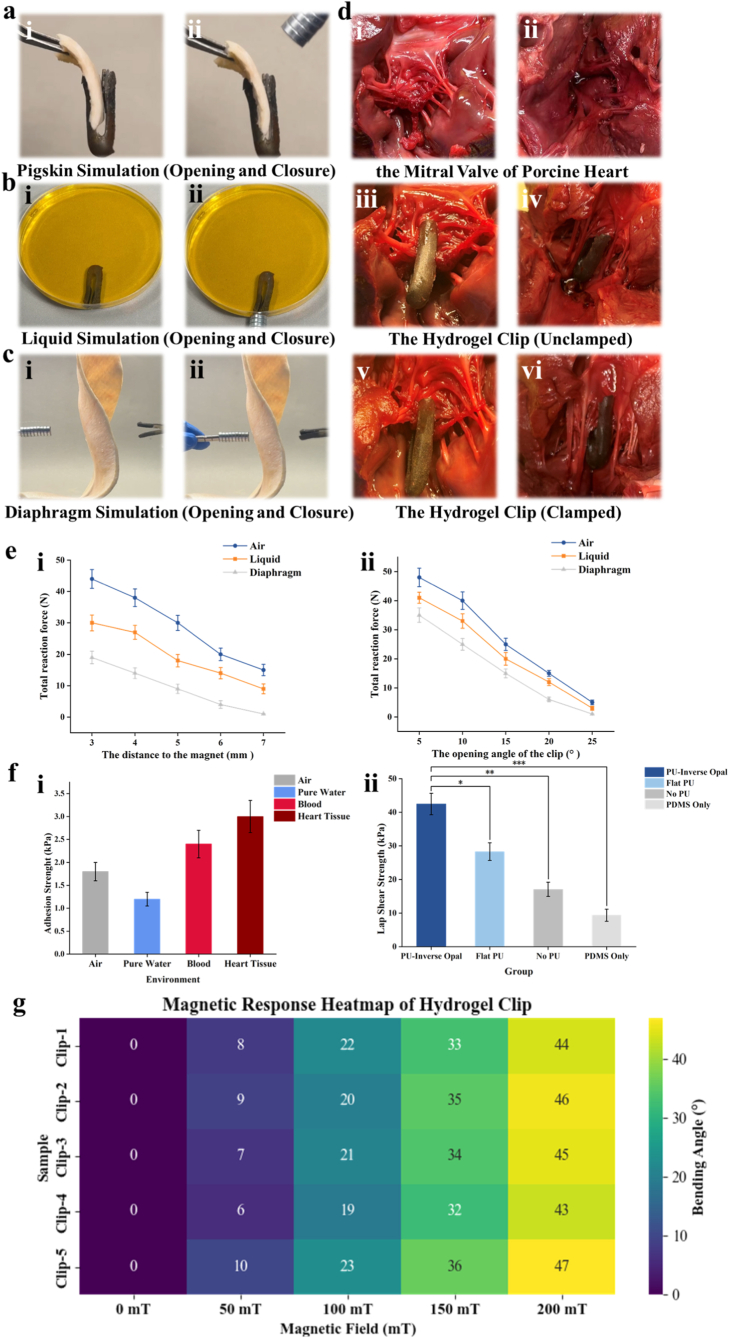
Demonstration of the magnetic actuation, functional performance relevance of the Y-shaped hydrogel clip across multiple simulation and physiological models. (a) Ex vivo simulation using porcine skin in air: (i) the initial undeformed configuration of the Y-shaped hydrogel clip shows its bifurcated arms ready for actuation; (ii) upon application of an external magnetic field, the arms of the clip undergo rapid bending, mimicking a clamping motion, highlighting its responsiveness and structural integrity in air. (b) Ex vivo simulation in a fluidic environment using porcine skin submerged in liquid: (i) undeformed initial state of the clip; (ii) the clip exhibits a similar magnetically induced bending deformation under fluidic resistance, demonstrating its applicability in dynamic biological environments such as blood flow or interstitial fluid. (c) Diaphragm-like flexible membrane model simulating thin tissue or organ walls: (i) initial flat morphology of the hydrogel clip; (ii) magnetic actuation results in a stable deformation, validating the device's performance across substrates with different mechanical compliances. (d) In vivo application in porcine mitral valve implantation: (i, ii) anatomical visualization of the mitral valve leaflets from two different perspectives prior to intervention; (iii, iv) the Y-shaped hydrogel clip is delivered through the venous system and magnetically guided to the targeted mitral valve location, demonstrating accurate navigation under magnetic control; (v, vi) upon magnetic activation, the clip firmly clamps the mitral leaflets, confirming the feasibility of non-invasive, remote-controlled implantation for cardiac repair applications. (e) Quantitative mechanical evaluation under magnetic fields: (i) the total reactive force exerted by the hydrogel clip during actuation is plotted against magnet distance in three distinct conditions (air, liquid, membrane), revealing consistent force generation with environmental adaptation; (ii) corresponding changes in the opening-closing angle of the clip under different environments, further supporting the robust mechanical adaptability of the design. (f) Assessment of adhesion and interfacial mechanical integrity: (i) Bar graph comparing the adhesion strength of the Y-shaped hydrogel clip in four distinct environments: air, pure water, blood, and cardiac tissue. The clip exhibited the highest adhesion strength in the cardiac tissue model, indicating superior conformability and integration with soft, dynamic biological substrates. (ii) Comparison of lap shear strength among clips fabricated with different material compositions, demonstrating that the optimized formulation significantly outperforms the control groups in resisting interfacial shear stress under physiological loading conditions. Data are presented as mean ± SD. Statistical analysis was performed using one-way ANOVA. ∗p < 0.05, ∗∗p < 0.01, ∗∗∗p < 0.001; data presented as mean ± SD; n = 5 per group. (g) Heatmap illustrating the angular response of different Y-shaped hydrogel clips under various magnetic field strengths: each cell represents the average actuation angle achieved by the clips at the corresponding magnetic intensity, demonstrating reliable and tunable deformation behavior in response to external magnetic stimulation.

To fully evaluate the magnetically actuated performance and functional feasibility of the Y-shaped hydrogel clip, we conducted a series of experiments on multiple in vitro simulation platforms and in vivo animal models, as shown in Fig. 5. These multi-scenario evaluations are designed to simulate different biological environments and evaluate the practical adaptability and reliability of the hydrogel clip under conditions that are very close to actual biomedical application conditions. In the first set of experiments (Fig. 5a), we used pig skin to perform in vitro simulations in an air environment to evaluate the basic structural response of the hydrogel clip under dry, resistance-free conditions. The initial bifurcated structure of the hydrogel clip (Fig. 5a–i) quickly transformed into a closed state under external magnetic stimulation (Fig. 5a–ii), demonstrating its rapid response and mechanical stability. This dry condition model provides a control reference for understanding the intrinsic responsiveness of magnetic hydrogel structures without the influence of fluid or tissue damping. Next, we evaluated the device under underwater conditions by immersing it in a liquid environment (Fig. 5b), which more closely simulates physiological conditions such as blood or interstitial fluid. Despite the increased resistance imposed by the surrounding fluid, the hydrogel clip maintained its ability to undergo magnetic bending deformation (Fig. 5b–ii), demonstrating reliable actuation in a submerged setting. The preservation of actuation dynamics in a viscous medium underscores the device's hydrodynamic adaptability, which is critical for its application in cardiovascular or other fluid-exposed interventional scenarios. We further tested the clip using a diaphragm-like flexible membrane model to simulate thin and deformable tissue surfaces (Fig. 5c). In this environment, the clip exhibited stable magnetically triggered actuation (Fig. 5c–ii), enhancing its ability to effectively operate on flexible substrates such as organ walls or vascular tissue. The successful performance in these different mechanical environments highlights the clip's robust design and broad translational potential. To verify in vivo feasibility, we used a porcine mitral valve model (Fig. 5d). Prior to implantation, the mitral valve leaflets were observed from two anatomical angles (Fig. 5d–i-ii). The Y-shaped hydrogel clip was delivered intravenously and positioned to the desired leaflet attachment points (Fig. 5d**,** iii-iv) by magnetic navigation, demonstrating its ability to achieve precise and minimally invasive positioning via remote magnetic navigation. After activation, the hydrogel clip firmly clamped the mitral valve leaflets (Fig. 5d–v-vi), confirming the potential of this system as a non-invasive alternative to existing transcatheter mitral valve repair device. This result is significant because it demonstrates the possibility of remote-controlled, suture less valve repair surgery. To quantitatively evaluate the mechanical properties, we measured the total reaction force generated by the hydrogel clip under magnetic actuation in three environments (air, liquid, and diaphragm) (Fig. 5e–i). Under all conditions, the actuation force showed a significant downward trend with increasing magnet distance, among which the resistance of the diaphragm model was slightly higher, which may be due to its elastic compliance. These results show that the clip is able to generate sufficient clamping force under different mechanical loads. Correspondingly, its opening and closing angle (Fig. 5e–ii) showed environment-dependent regulatory characteristics, further demonstrating the adaptability of the clip and its sensitivity to environmental mechanical constraints. Magnetic safety window and site-specific actuation. In addition to demonstrating controllable actuation in the forward-field direction, clinical deployment of magnetic implants requires clear evidence that reverse magnetic perturbations will not inadvertently trigger device opening, and that activation remains specific to the target site even in the presence of potential magnetic interference. Therefore, we performed a reverse-field mis-activation threshold test in PBS to mimic hydrated physiological media (e.g., blood or interstitial fluid). As illustrated in Fig. S5(a), the clip was first set to a stable closed working state, after which an external magnetic field oriented opposite to the normal actuation direction was applied as a worst-case disturbance. A linear Hall sensor placed adjacent to the clip continuously calibrated the local magnetic flux density B, and the magnet–clip distance was gradually reduced to identify any sustained, discernible reopening displacement or functional release. The minimal local field that caused functional unclamping was defined as the reverse mis-activation threshold $B_{\text{th},\text{reverse}}$. The Hall-calibrated distance–field relationship (Fig. S5(b)) shows that the clip remained stably closed under reverse fields and only exhibited reopening when $B_{\text{th},\text{reverse}}=95\;\text{mT}$ (n = 6), which is substantially higher than the forward actuation threshold required for normal opening, $B_{\text{th},\text{open}}=35\;\text{mT}$. This yields a safety margin of $B_{\text{th},\text{reverse}}/B_{\text{th},\text{open}}=2.7$, indicating strong direction selectivity and a quantifiable reverse-field safety window. Consistently, the opening-angle response versus local flux density (Fig. S5(c)) confirms that effective opening occurs only above $B_{\text{th},\text{open}}$ under forward fields, whereas reverse-field reopening is not triggered until $B_{\text{th},\text{reverse}}$ is reached. Together with the rapid spatial decay of magnetic field strength, this safety window implies that low-intensity ambient fields or off-target magnetic sources are unlikely to reach the effective actuation threshold at non-target locations, thereby supporting site-specific activation. We further note that extreme magnetic exposure scenarios (e.g., MRI proximity or strong clinical magnetic equipment) warrant additional system-level and in vivo safety evaluations, which will be addressed in future work. Adhesion strength (Fig. 5f–i) was quantitatively assessed under four different environmental conditions: air, pure water, blood, and ex vivo cardiac tissue. Among these, the Y-shaped hydrogel clip exhibited the highest adhesion strength on cardiac tissue, likely attributable to its enhanced surface conformability and intimate contact with the moist, elastic myocardial substrate. This superior adhesive performance underscores the device's potential for stable fixation in dynamic, soft tissue environments, which is critical for cardiovascular interventions. Furthermore, lap shear strength evaluations (Fig. 5f–ii) comparing different material formulations revealed that the optimized hydrogel composition significantly outperformed the controls, providing enhanced interfacial mechanical robustness. When combined with the graph showing the change in adhesion force over time (Fig. S2), it can be seen that different materials (PU - Inverse Opal, Flat PU, NO PU, PDMS Only) exhibit different trends in adhesion force over time. Among them, the material with the PU - Inverse Opal structure has a relatively high adhesion force and a small decrease, while the adhesion force of other materials decreases more significantly over time. These results validate the design and material strategy employed, ensuring both mechanical durability and reliable tissue anchoring during in - vivo deployment. Importantly, while the above adhesion and lap-shear tests confirm robust static fixation, stable grasping under continuous rhythmic cardiac motion is equally critical. Therefore, we further conducted an in vitro high-frequency cyclic pressure/flow impact test to approximate pulsatile hemodynamic loading during the cardiac cycle. Specifically, a high-pressure split-chamber solenoid-valve drainage system was constructed to generate rapid fluid impulses every 0.5 s (≈120 bpm) **(**Video S3**)**, thereby applying continuous rhythmic loading to the hydrogel clip pre-attached to a leaflet-mimicking model. After 100,000 cycles, we observed (1) no direct detachment of the clip and (2) no noticeable slippage, as indicated by the unchanged position of the clamping-region markers (Fig. S4). These results demonstrate reliable short-term dynamic clamping stability of the device under physiologically relevant cyclic fluid impacts. We note that longer-term fatigue and dynamic stability validation (10^5^–10^6^ cycles) in a full left-heart pulsatile simulator or in vivo models remains to be completed and will be addressed in future work to further assess safety and reliability under prolonged cyclic conditions. Finally, the angular deformation response under different magnetic field intensities was visualized using a heatmap (Fig. 5g). Across multiple samples, consistent and progressively increased bending angles were recorded as magnetic intensity rose, demonstrating the repeatable and tunable nature of the device's magnetic actuation. This reliable control over deformation amplitude is essential for tailoring the clip's performance to specific anatomical and therapeutic requirements. In conclusion, the integrated findings across simulation platforms and live animal models provide compelling evidence for the functional robustness, mechanical reliability, and biomedical applicability of the magnetically responsive Y-shaped hydrogel clip. These results establish a solid foundation for future translational exploration in minimally invasive cardiovascular and tissue repair procedures. To establish an objective and measurable definition of “successful clipping,” we quantified the coaptation length (CL) after device deployment and incorporated it into our success criteria. After the clip was positioned and the valve/leaflet model was brought to a closed state, images were captured from the valvular orifice direction with a calibrated scale included in the field of view. CL was defined as the overlap length between the free edges of the anterior and posterior leaflets along the normal coaptation direction after clipping. High-resolution images were analyzed in ImageJ; for each sample, CL was measured at three representative locations along the coaptation line and averaged to obtain one CL value per sample (n≥5). The revised Results now include quantitative CL statistics, and achieving a predefined CL threshold (together with sufficient clamping force and absence of noticeable slippage) was used as one of the measurable criteria for successful leaflet approximation (Fig. S6).

To comprehensively characterize the mechanical performance of the magnetically responsive hydrogel clip, we introduce a coupled theoretical framework that integrates nonlinear elasticity, magnetic field theory, and multilayer tissue interactions. The total traction force $F_{t}$ generated by the hydrogel actuator can be expressed as a function of the internal stress-strain response of the hydrogel and the external magnetic field gradient. The magnetic force acting on the superparamagnetic domains embedded in the hydrogel follows:

(1) $$\begin{array}{c} F_{m}=\mu_{0}\;\int_{V}\left(M\cdot \nabla \right)\text{Hdv} \end{array}$$

where M is the magnetization vector within the hydrogel matrix, and H is the spatial magnetic field generated by the external permanent magnet. For a uniformly magnetized actuator with magnetic susceptibility χ, and in the presence of a dipole-like field from a cylindrical neodymium magnet, the field intensity at axial distance z can be approximated by:

(2) $$\begin{array}{c} H\left(z\right)=\frac{B_{r}}{2\mu_{0}}\left(\frac{L}{{\left(z^{2}+R^{2}\right)}^{3/2}}\right) \end{array}$$

Here, $B_{r}$ is the remanent magnetic flux density of the magnet, L and R are its length and radius, respectively. The resulting axial force on the actuator then becomes a highly nonlinear function of distance z, which aligns with the experimental force-distance trend shown in (Fig. 5e–i). As the magnet approaches, the increase in field gradient leads to a rapid rise in magnetic traction, consistent with the curve profile predicted by this model.

In modeling the mechanical resistance of the hydrogel arms during actuation, we adopt the Gent hyperelastic model, which accounts for strain stiffening near the polymer chain extensibility limit:

(3) $$\begin{array}{c} W=-\frac{\mu J_{m}}{2}\ln\left(1-\frac{I_{1}-3}{J_{m}}\right) \end{array}$$

where W is the strain energy density, μ is the shear modulus, $J_{m}$ is a material constant representing the limiting chain extensibility, and $I_{1}$ is the first invariant of the deformation tensor. This model more accurately reflects the stiffening behavior of the hydrogel under large deformations during clip closure. The reaction force $F_{\text{elastic}}$ can be obtained by differentiating the total potential energy with respect to deformation λ:

(4) $$\begin{array}{c} F_{\text{elastic}}\left(\lambda \right)=A\cdot \frac{dW}{d\lambda } \end{array}$$

Where A is the effective cross-sectional area and λ the stretch ratio. The dynamic balance between $F_{\text{elastic}}$ and ${\;F}_{m}$ governs the real-time deformation behavior of the clip, reflected by the angular displacement in (Fig. 5e–ii), which shows the experimentally measured opening angle as a function of magnet distance. Finally, for actuation across multilayer tissue barriers, we incorporate a correction factor $\alpha_{eff}$ to account for the viscoelastic damping by surrounding tissue:

(5) $$\begin{array}{c} F_{transmitted}=\alpha_{eff}\left(d,E_{t},\nu_{t}\right)\cdot F_{m} \end{array}$$

where d is the diaphragm thickness, and $E_{t}$, $\nu_{t}$ are the Young's modulus and Poisson's ratio of the tissue-mimicking diaphragm. The correction factor $\alpha_{eff}$ can be approximated from classical plate theory for small deflections:

(6) $$\begin{array}{c} \alpha_{eff}\approx {\left(1+\frac{12\left(1-\nu_{t}^{2}\right)d^{2}}{h^{2}}\right)}^{-1} \end{array}$$

where h is the lateral contact dimension between the hydrogel and the diaphragm. This factor predicts a mechanical filtering effect, where only partial magnetic force is transmitted, aligning with the decreased traction observed experimentally. To resolve the force distribution on embedded superparamagnetic domains during clip: deformation, we model each particle i as a magnetic dipole within an Updated-Lagrangian (or ALE) description. The particle position $x_{i}$ is mapped from its reference coordinate $X_{i}$ by the deformation mapping φ:

(7) $$\begin{array}{c} x_{i}\left(t\right)=\varphi \left(X_{i},t\right),F\left(X_{i},t\right)=\nabla x\varphi \end{array}$$

The domain magnetization at the particle follows the local material rotation (polar decomposition F=RU)

(8) $$\begin{array}{c} M_{i}\left(t\right)=R\left(F\left(X_{i},t\right)\right)M_{0,i}, \end{array}$$

with dipole moment $m_{i}=V_{i}M_{i}$ ($V_{i}$: particle volume). The magnetic force and torque on particle i in the current magnetic field B(x,t) are

(9) $$\begin{array}{c} F_{i}=\nabla \left(m_{i}\cdot B\right){,|}_{x_{i}},\tau_{i}=m_{i}\times B\left(x_{i}\right) \end{array}$$

The particle forces are assembled into an equivalent magnetic body force density acting on the hydrogel continuum,

(10) $$\begin{array}{c} f_{mag}\left(x,t\right)=\sum_{i}F_{i}N_{i}\left(x\right) \end{array}$$

Where $N_{i}\left(x\right)$ are element shape functions that distribute the nodal loads in the updated configuration. The hydrogel equilibrium (nonlinear elasticity) at each updated step then reads

(11) $$\begin{array}{c} \nabla \cdot P\left(F\right)+f_{mag}=0 \end{array}$$

Equations (7), (8), (9), (10), (11) are solved in a staggered manner (magnetic field on the updated geometry, particle forces by Eq. (9), structural update by Eq. (11)), ensuring that the domain orientations $M_{i}$, their relative positions $x_{i}$, and the resulting magnetic force distribution evolve consistently with clip deformation.

Simplified pulsatile mitral regurgitation model and quantification of regurgitant low/EROA reduction. To quantitatively assess the functional efficacy of the Y-shaped hydrogel clip in reducing mitral regurgitation (MR) under an edge-to-edge repair strategy, we established a syringe-driven simplified pulsatile MR model and measured regurgitant volume and effective regurgitant orifice area (EROA) before and after clipping. Fresh porcine mitral leaflets (or intact ex vivo mitral valves) were mounted between two custom transparent chambers representing the left ventricular (LV) and left atrial (LA) sides. Leaflet edges were sutured and secured to a circular annulus to preserve native coaptation geometry. Functional regurgitation was induced by controlled shortening of the posterior chordae tendineae (∼3–5 mm) until a stable reverse backflow was observed under cyclic pressure loading. The Y-shaped hydrogel clip was then applied in an edge-to-edge manner to grasp both anterior and posterior leaflets; correct clamping position was visually confirmed, and incompletely clamped samples were excluded.

Because ultrasound and a full pulsatile left-heart simulator were not available at this proof-of-concept stage, a syringe-driven pulsatile leakage model was used to generate physiologically relevant LV–LA pressure gradients. A 100-mL syringe was connected to the LV chamber via tubing, and piston push–pull cycles were manually driven at 120 beats/min (2 Hz) using a metronome. Each piston push corresponded to systole (regurgitation phase), whereas piston pull/relaxation corresponded to diastole (regurgitation cessation). The instantaneous piston force F(t) was recorded using a handheld force gauge and extracted frame-by-frame from synchronized video to obtain a force–time waveform. The syringe inner diameter was measured as d=20 mm giving a piston cross-sectional area:

(12) $$\begin{array}{c} A=\pi {\left(\frac{d}{2}\right)}^{2}=\pi {\left(0.0145\right)}^{2}=6.60\times 10^{-4}\;m^{2} \end{array}$$

The LV pressure was calculated as:

(13) $$\begin{array}{c} P_{LV}\left(t\right)=\frac{F\left(t\right)}{A} \end{array}$$

The measured peak piston force was 9.2 ±1.1N, corresponding to a peak LV pressure:

(14) $$\begin{array}{c} P_{LV,peak}=\frac{9.2}{6.60\times 10^{-4}}=1.39\times 10^{4}\;Pa\approx 104\;mmHg \end{array}$$

Where 1 mmHg = 133.3 Pa. The LA baseline pressure was set using a vertical fluid column connected to the LA outlet. With a fluid height difference of h=13.6 cm, the LA pressure was:

(15) $$\begin{array}{c} P_{LA}=\rho gh=1000\times 9.81\times 0.136=1333\;Pa\approx 10\;mmHg \end{array}$$

where ρ = (PBS density) and g = 9.81 m/s. The transvalvular pressure gradient was defined as:

(16) $$\begin{array}{c} \Delta P\left(t\right)=P_{LV}\left(t\right)-P_{LA} \end{array}$$

For MR estimation, the peak ΔP during piston push (regurgitation phase) was averaged over 10 consecutive cycles. Under this condition, the peak transvalvular gradient was 95 ±12mmHg and was maintained identical before and after clipping.

Regurgitant volume was quantified by collecting reverse leakage into the LA chamber for 60 s under constant frequency and piston force, yielding the total regurgitant volume $V_{reg,total}$. At 120 bpm, the number of beats in 60 s was: $N_{beats}=120\text{.}$ The regurgitant volume per beat was calculated as:

(17) $$\begin{array}{c} V_{reg/beat}=\frac{V_{reg,total}}{N_{beats}} \end{array}$$

The mean regurgitant flow rate was:

(18) $$\begin{array}{c} Q_{reg}=V_{reg/beat}\times HR \end{array}$$

and was converted into m^3^/s prior to EROA estimation. In the absence of ultrasound, EROA was estimated using an orifice equation:

(19) $$\begin{array}{c} REROA=\frac{Q_{reg}}{C_{d}\sqrt{\frac{2\Delta P}{\rho }}} \end{array}$$

with a discharge coefficient $C_{d}$ = 0.7 and ρ = 1000 kg/m^3^.A sensitivity analysis with $C_{d}$ = 0.6–0.8 was performed to confirm trend robustness.

For each sample, regurgitant volume and EROA were measured before clipping (pre-clip) and after clipping (post-clip) under identical loading conditions. Percentage reductions were calculated as:

(20) $$\begin{array}{c} \Delta V_{reg}=\frac{V_{reg/beat,pre}-V_{reg/beat,post}}{V_{reg/beat,pre}}\times 100\%,\Delta EROA=\frac{EROA_{pre}-EROA_{post}}{EROA_{pre}}\times 100\% \end{array}$$

Six independent samples were tested (n=6); data are presented as mean ± SD, and pre-versus post-clip values were compared using paired t-tests.

### 3.4 In vitro blood compatibility and anticoagulant performance

To evaluate the hemocompatibility and anticoagulant properties of the tested materials, namely the Positive (glass slide), commercial MitraClip, and the Y-Shaped hydrogel clip, comprehensive in vitro experiments were conducted. These included whole blood clotting time, plasma recalcification time, relative hemolysis ratio, and platelet adhesion assessments. The protocols involving rats and New Zealand white rabbits were approved by the Animal Ethics Committee of a hospital in Shanghai, ensuring compliance with institutional and national ethical guidelines.

**Fig. 6:**
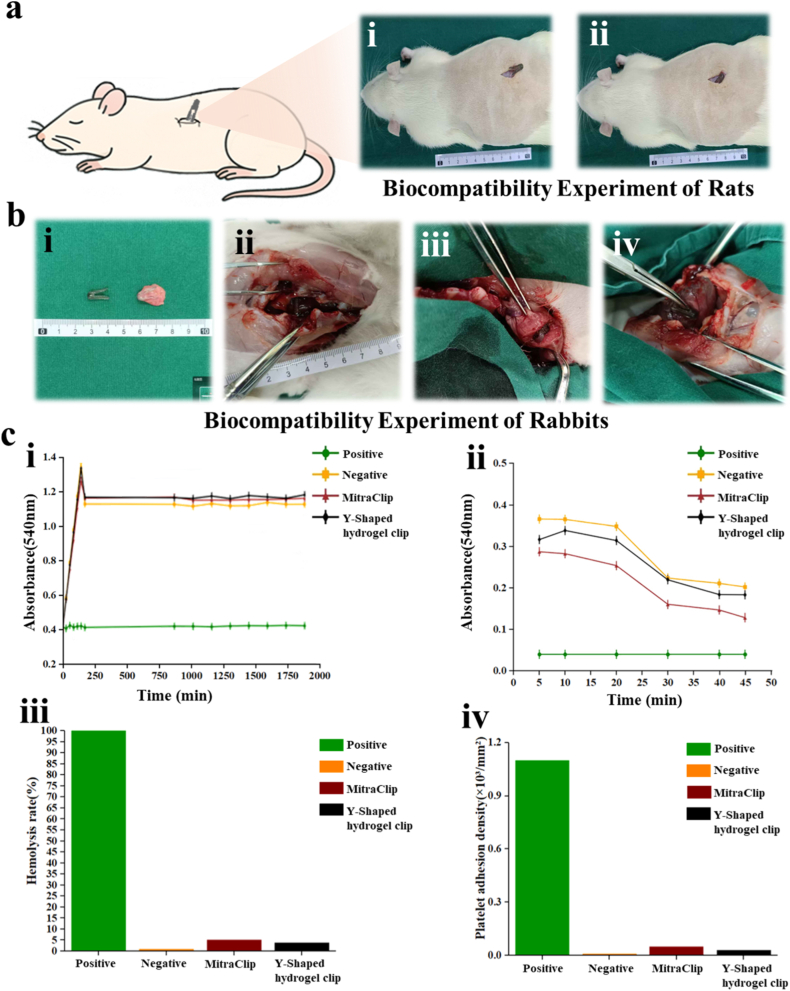
In vivo biocompatibility evaluation of the Y-shaped hydrogel clip. (a) Schematic and photographic illustrations of the subcutaneous implantation of the hydrogel clip in rats. (i–ii): External view of the implantation site post-surgery showing the position of the hydrogel clip under the skin. (b) Representative intraoperative images illustrating animal experiments. (i) Hydrogel clip and excised tissue prior to implantation in the rat model, used to assess basic biocompatibility. (ii–iv) Stepwise placement of the hydrogel clip adjacent to the rabbit heart within the thoracic cavity, demonstrating magnetic actuation in a pulsatile environment rather than functional mitral valve repair. (c) (i) Whole blood clotting time evaluation showing rapid hemostatic behavior compared to control groups (n = 5). (ii) Plasma recalcification time demonstrating improved coagulation compatibility of the hydrogel clip (n = 5). (iii) Quantitative analysis of relative hemolysis rate, confirming low hemolytic activity and favorable blood compatibility of the 3D printed hydrogel clip (n = 5). (iv) Quantification of lactate dehydrogenase (LDH) activity as an indicator of cytotoxicity, with results showing minimal LDH release, comparable to the negative control (n = 5). All data are presented as mean ± standard deviation (SD). ∗p < 0.05, ∗∗p < 0.01.

To preliminarily assess the implantation performance and tissue tolerance of the Y-Shaped hydrogel clip, subcutaneous and thoracic cavity implantations were conducted in Sprague-Dawley rats. As shown in (Fig. 6a), schematic and photographic illustrations depict the subcutaneous placement of the hydrogel clip beneath the dorsal skin. Postoperative external views (Fig. 6a–i–ii) confirm the precise positioning of the device without inducing visible inflammation or tissue disruption. Further, representative intraoperative images in (Fig. 5b) illustrate the surgical procedure for thoracic implantation. Image (Fig. 6b–i) presents the excised thoracic tissue and the hydrogel clip prior to insertion. Sequential images (Fig. 6b, ii–iv) show the stepwise implantation and fixation process within the thoracic cavity, demonstrating the conformal integration of the hydrogel clip with the target tissue. These visual observations support the material's surgical manipulability, mechanical resilience, and tissue compatibility, laying the foundation for its clinical translational potential. The whole blood clotting assay provides an overview of the material's influence on coagulation kinetics. After incubation with activated rabbit blood, the absorbance of the supernatant at 540 nm, indicative of free hemoglobin concentration, was monitored over time. As shown in (Fig. 6c–i), the Positive group exhibited the highest absorbance at all time points, indicating limited coagulation and a longer clotting time. In contrast, both the MitraClip and Y-Shaped hydrogel clip groups demonstrated significantly lower absorbance values, suggesting enhanced clot formation. Notably, the presence of IC-functionalized SiO_2_ nanoparticles on the surface did not adversely affect coagulation. The comparable performance between the MitraClip and hydrogel clip implies that the modification preserves clotting efficiency while potentially improving safety. These results suggest that surface nanostructures and superhydrophobic coatings contribute to a balanced coagulation response—avoiding both thrombosis and excessive bleeding—essential for cardiovascular implants. The plasma recalcification assay was used to evaluate the material's interaction with the intrinsic coagulation pathway. Upon reintroducing Ca^2+^ to citrated platelet-poor plasma, clot formation was tracked at 405 nm. The Positive group showed an inflection point at approximately 20 min, while the positive control (CaCl_2_-activated plasma) coagulated within 15 min, as expected. However, both the MitraClip and Y-Shaped hydrogel clip groups maintained minimal changes in absorbance over the full 45-min test duration, closely resembling the negative control (non-recalcified plasma), as shown in (Fig. 6c–ii). This lack of an inflection point indicates negligible intrinsic pathway activation, which is highly desirable for blood-contacting materials. The results suggest that the superhydrophobic surface introduced by the uniformly dispersed IC-SiO_2_ nanoparticles inhibits plasma protein adsorption and coagulation factor activation, thereby reducing thrombogenic potential. Moreover, the antibacterial agent IC did not compromise this anticoagulant behavior, underscoring the stability and functionality of the coating. Hemolysis evaluation is critical in determining whether a material causes mechanical or chemical damage to red blood cells. According to ISO 10993-4 standards, materials with hemolysis ratios below 5 % are considered blood-compatible. In this study, the Positive group exhibited a hemolysis ratio of 3.418 %, which is within the acceptable range. After coating with SiO_2_ nanoparticles, the MitraClip's hemolysis ratio decreased to 2.016 %, while the Y-Shaped hydrogel clip demonstrated the lowest ratio of 1.967 % (Fig. 6c–iii). The pronounced improvement in hemocompatibility highlights the role of the nanostructured superhydrophobic surface in minimizing direct RBC contact and rupture. These findings confirm that both the base material and the functional coating are inherently safe for blood-contacting applications, further reinforcing the suitability of the Y-Shaped hydrogel clip for clinical use. Platelet adhesion and subsequent activation are key indicators of thrombogenic risk. After incubation with platelet-rich plasma, SEM imaging and quantitative LDH assay revealed substantial differences among the tested materials (Fig. 6c–iv). The Positive group exhibited dense platelet coverage (∼1249 platelets/mm^2^), consistent with high thrombogenicity. In contrast, the MitraClip and Y-Shaped hydrogel clip had significantly reduced platelet adhesion densities of 102 and 98 platelets/mm^2^, respectively. These results suggest that the superhydrophobic surface effectively repels platelet adhesion and inhibits activation. Furthermore, the presence of the antibacterial IC compound in the coating did not negatively impact antiplatelet performance, thereby preserving both antimicrobial and antithrombotic functions. This dual functionality is highly advantageous for cardiovascular implants, where both infection prevention and hemocompatibility are critical.

In summary, the Y-Shaped hydrogel clip demonstrates excellent in vitro blood compatibility and anticoagulant properties across multiple standardized assays. Compared to both the unmodified Positive and the clinically used MitraClip, the hydrogel clip exhibited superior performance in minimizing hemolysis, reducing platelet adhesion, and inhibiting intrinsic coagulation activation. These enhancements can be attributed to the tailored superhydrophobic nanostructure and the multifunctional IC-SiO_2_ nanoparticle surface, which jointly reduce protein adsorption and cellular interaction without compromising pro-coagulant behavior. Collectively, these features establish the Y-Shaped hydrogel clip as a promising candidate for safe, long-term implantation in cardiovascular applications.

## 4 Conclusion

In this study, we successfully developed a magnetically responsive Y-shaped hydrogel mitral clip incorporating hierarchical inverse opal photonic crystal structures and Janus wettability for minimally invasive cardiac valve repair. The clip is composed of a photopolymerized GelMA/Fe_3_O_4_ composite hydrogel core for magnetic actuation, a mechanically compliant epoxy acrylate intermediate scaffold, a biocompatible PDMS outer interface, and an outermost polyurethane (PU) layer engineered with an inverse opal architecture. This PU layer enhances tissue adhesion and provides mechanical protection, while also contributing to the Janus wettability design—hydrophilic on the inner side to promote tissue anchoring and hydrophobic on the blood-contacting outer side to suppress thrombogenesis. Spatially controllable deformation under external magnetic fields was validated through in vitro, ex vivo, and COMSOL-based simulation studies, confirming remote actuation and robust tissue clamping. Notably, the Y-shaped clip demonstrated stable magnetic navigation and deployment within the mitral valve region in a porcine heart model. Mechanical tests confirmed sustained performance under cyclic deformation and physiological stress conditions. Blood compatibility evaluations—including whole blood clotting time, plasma recalcification, hemolysis ratio, and platelet adhesion density—revealed markedly superior anticoagulant properties compared to commercial MitraClip and other control materials. Furthermore, the device retained its mechanical and biological function even after integration of photonic and adhesive components.

Overall, this study presents a compliant, magnetically actuated GelMA hydrogel valve clip that integrates minimally invasive edge-to-edge deployment, conformal leaflet engagement, microstructure-enhanced wet adhesion, and encouraging hemocompatibility—thereby suggesting a feasible soft-material alternative within the TEER framework. Nevertheless, we emphasize that the current evidence remains at a proof-of-concept level and must be interpreted within clear boundaries. First, the present experiments were conducted in simplified ex vivo and bench-top settings; a fully enclosed, anatomically accurate pulsatile left-heart simulator capable of reproducing annular dynamics and three-dimensional flow fields has not yet been implemented, which limits direct extrapolation to in vivo cardiac environments. Second, although we demonstrated significant reductions in regurgitant volume/EROA and stable coaptation restoration under controlled conditions, these findings require confirmation in higher-fidelity physiological models, particularly under long-term hemodynamic loading and tissue remodeling. Third, while our reverse-field threshold tests establish a quantifiable magnetic safety window in wet media, system-level magnetic exposure limits and operational considerations—especially for extreme scenarios such as MRI—remain to be defined. Finally, transcatheter deliverability and procedural operability in clinically realistic anatomies have not yet been evaluated and represent a key translational requirement.

To address these limitations, our next steps will include extended-duration fatigue and dynamic stability testing in advanced pulsatile left-heart simulators, acute and chronic large-animal (porcine) TEER studies to assess performance within true cardiac kinematics, blood-flow shear, and interface remodeling, and dedicated magnetic-safety evaluations across clinically relevant exposure envelopes, including MRI-related conditions, together with refinement of catheter-based delivery. These investigations are essential to bridge the gap between the present proof-of-concept results and eventual clinical translation, and to more precisely define the comparative advantages and boundaries of this soft-magnetic TEER strategy relative to rigid next-generation mechanical devices.

## CRediT authorship contribution statement

**Yue Wang:** Writing – original draft, Investigation. **Ximing Liao:** Writing – original draft, Investigation. **Lei Zhou:** Investigation. **Songchao Fu:** Investigation. **Qing He:** Data curation. **Xinqi Chen:** Validation. **Linxi Xia:** Formal analysis, Data curation. **Cihui Liu:** Writing – review & editing, Conceptualization. **Feng Liu:** Writing – review & editing, Conceptualization. **Lei Yang:** Writing – review & editing.

## Ethics approval and consent to participate

All animal experiments were conducted in accordance with a protocol approved by the Animal Care and Use Committee of Shanghai Sixth People's Hospital affiliated with Shanghai Jiao Tong University School of Medicine (Shanghai, China, number: 20210792). The study was conducted in accordance with the “3R” principle (reduction, replacement, and refinement).

## Declaration of competing interest

The authors declare no competing financial interests.

## Data Availability

All data needed to evaluate the conclusions in the paper are present in the paper and/or the Supplementary Materials.
