# Supplementary material for: Innovative Y-shaped hydrogel mitral clip with magnetic actuation and hydrophilic janus surface for enhanced valve leaflet repair
Source: Mater Today Bio. 2025 Dec 30;36:102750. doi: 10.1016/j.mtbio.2025.102750 (PMC12813318; doi:10.1016/j.mtbio.2025.102750)
Supplement: Multimedia component 1 [file mmc1.docx]

Supporting Information

**Innovative Y-Shaped Hydrogel Mitral Clip with Magnetic Actuation and Hydrophilic Janus Surface for Enhanced Valve Leaflet Repair**

*Yue Wang*,*^a^*^†^ *Ximing Liao*,*^b^*^†^ *Lei Zhou, ^c^*^†^ *Songchao Fu*, *^a^ Qing He*, *^a^ Xinqi Chen*, *^a^ Linxi Xia, ^a^ Cihui Liu*,*^a^* Feng Liu*,*^d^* Lei Yang^e*^*

a Center for Future Optoelectronic Functional Materials, School of Computer and Electronic Information/School of Artificial Intelligence, Nanjing Normal University, Nanjing, 210046, China.

b Department of Pulmonary and Critical Care Medicine, Shanghai East Hospital, School of Medicine, Tongji University, Shanghai, 200092, China.

c Department of Nursing, Shanghai General Hospital, Shanghai Jiao Tong University School of Medicine, Shanghai, China.

d Department of Nephrology, Shanghai East Hospital, School of Medicine, Tongji University, Shanghai, 200092, China.

e Department of Cardiology, Shanghai Children's Medical Center, Shanghai Jiao Tong University School of Medicine, Shanghai, 201600, China.

†These authors contributed equally to this work.

*Corresponding Authors:

cihui@njnu.edu.cn, [liufeng0113@126.com](mailto:liufeng0113@126.com), [yanglei403109@163.com](mailto:yanglei403109@163.com)


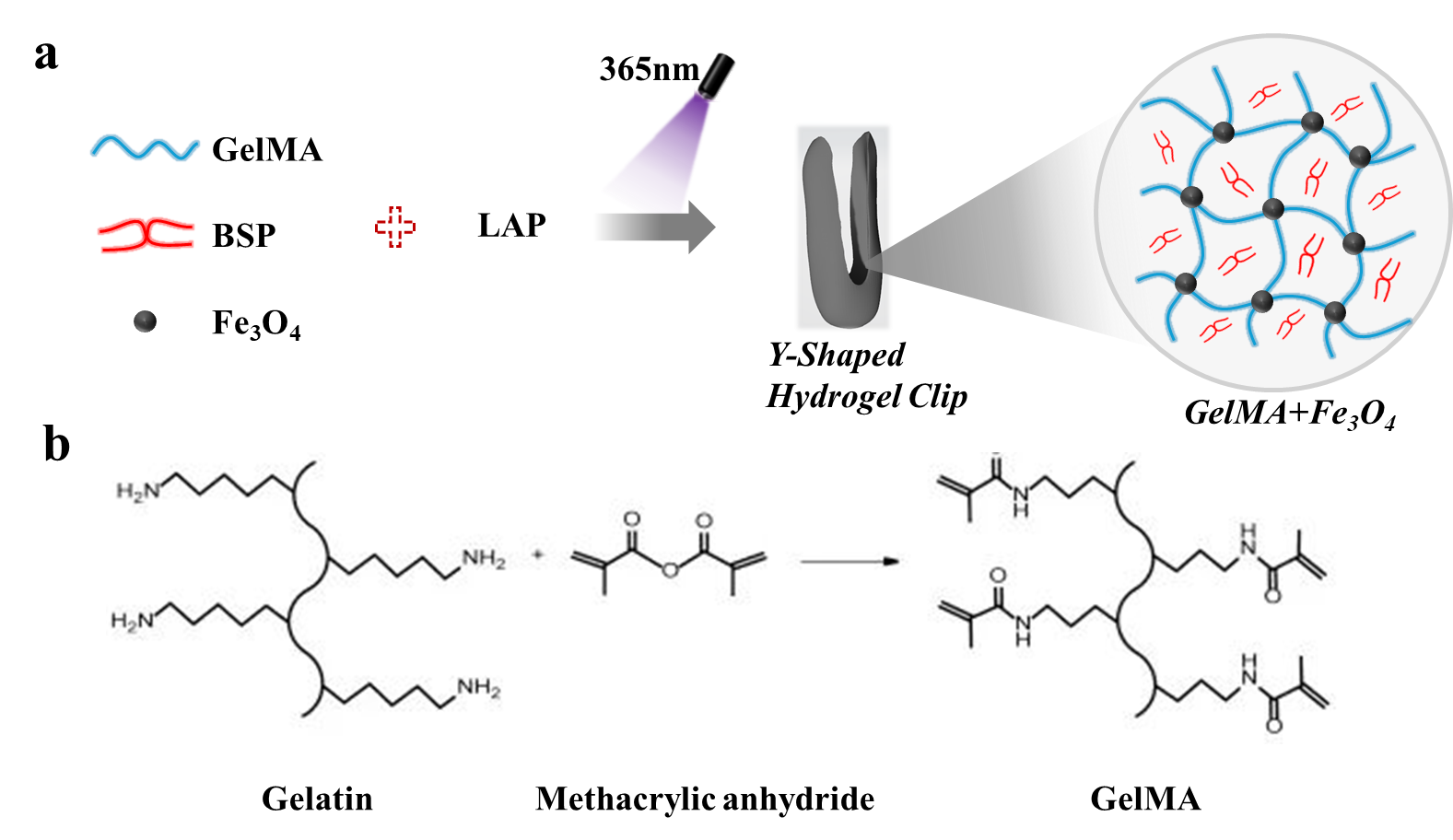


**Figure S1.** Schematic of GelMA/Fe_3_O_4_ Hydrogel Formation and Chemical Structure of GelMA (a) Schematic of GelMA/Fe_3_O_4_ hydrogel formation: Gelatin methacryloyl (GelMA, 10 wt%, methacrylation degree 85%) is dissolved in phosphate-buffered saline (PBS, pH 7.4) at 40°C, with lithium phenyl-2,4,6-trimethylbenzoylphosphinate (LAP, 0.5 wt%) as the photoinitiator and Fe_3_O_4_ nanoparticles (20 nm, 3 mg/mL) uniformly dispersed via ultrasonication (100 W, 10 min). The mixture is poured into a polydimethylsiloxane mold and exposed to 365 nm ultraviolet light (100 mW/cm²) for 20 s at 25°C, initiating free-radical crosslinking to form a magnetically responsive hydrogel. (b) Chemical structure of GelMA: Composed of gelatin backbone with methacryloyl groups introduced via reaction with methacrylic anhydride.


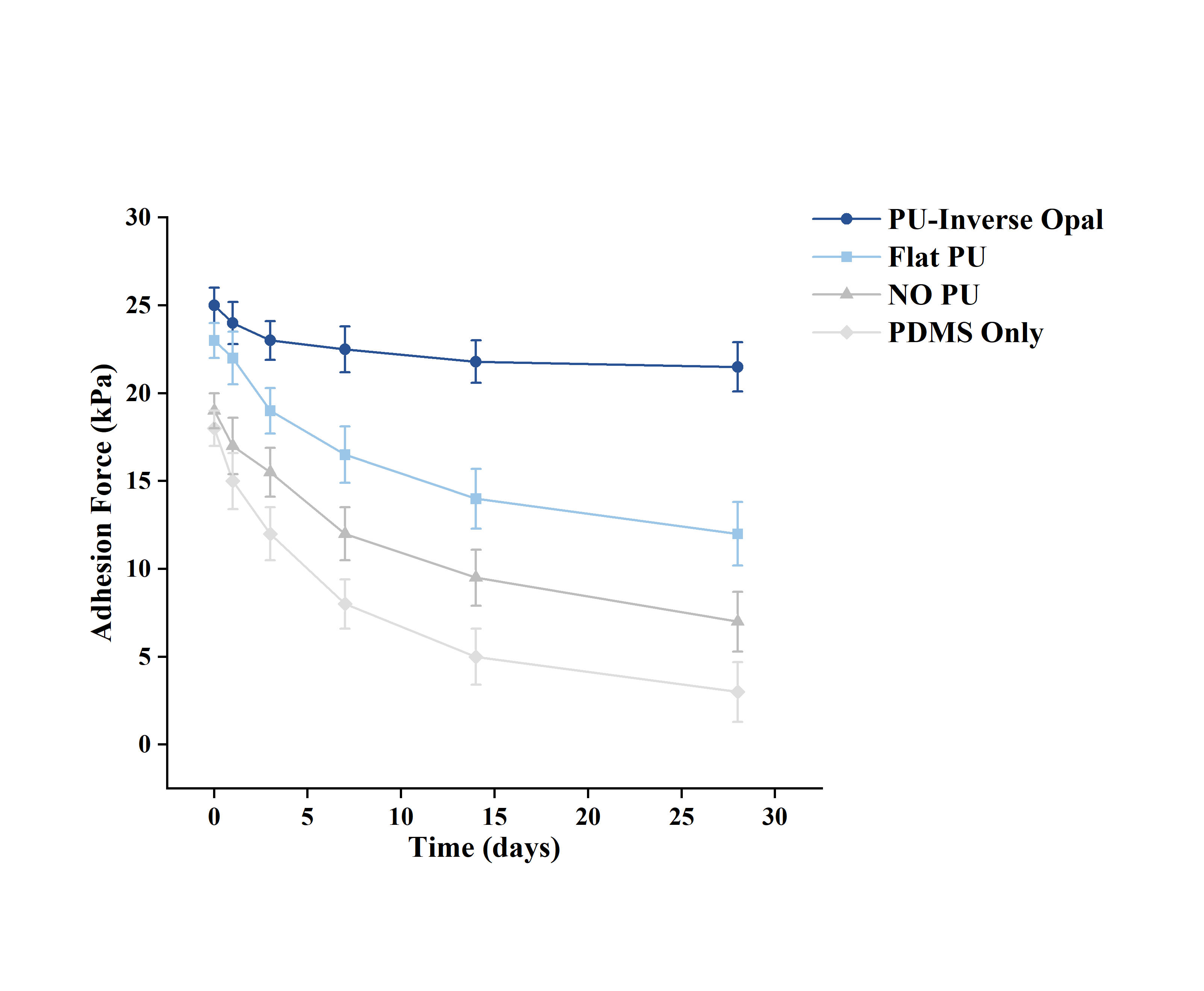


**Figure S2.** Comparison of the trends of adhesion force over time for different materials (PU - Inverse Opal, Flat PU, NO PU, PDMS Only).PU - Inverse Opal significantly outperforms the control groups.


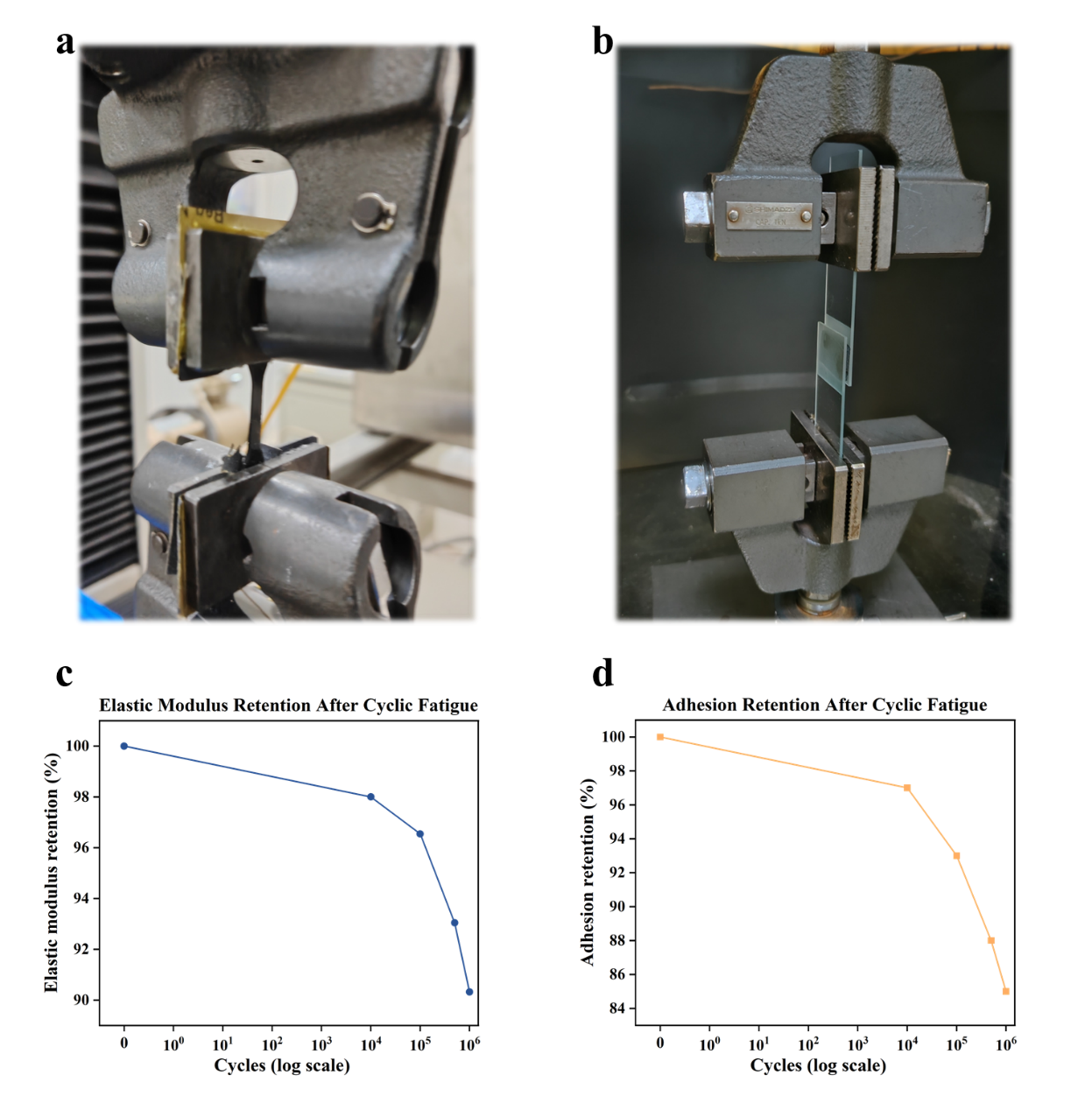


**Figure S3.** Mechanical and adhesive performance and cyclic durability of the Y-shaped hydrogel clip (a) Representative photograph of the tensile test setup for the Y-shaped hydrogel clip, used to evaluate its load–displacement response and elastic deformation stability under stretching. (b) Representative photograph of the adhesion test setup for the Y-shaped hydrogel clip, used to quantify interfacial adhesion strength under controlled contact/separation loading. (c) Elastic modulus retention of the clip after cyclic fatigue loading plotted versus cycle number (log scale), showing gradual but limited stiffness loss and indicating structural robustness under repeated mechanical deformation. (d) Adhesion retention of the clip after cyclic fatigue loading plotted versus cycle number (log scale), demonstrating that interfacial adhesion remains high over repeated cycles, with only moderate decay at high cycle counts, supporting long-term fixation reliability in dynamic physiological environments.


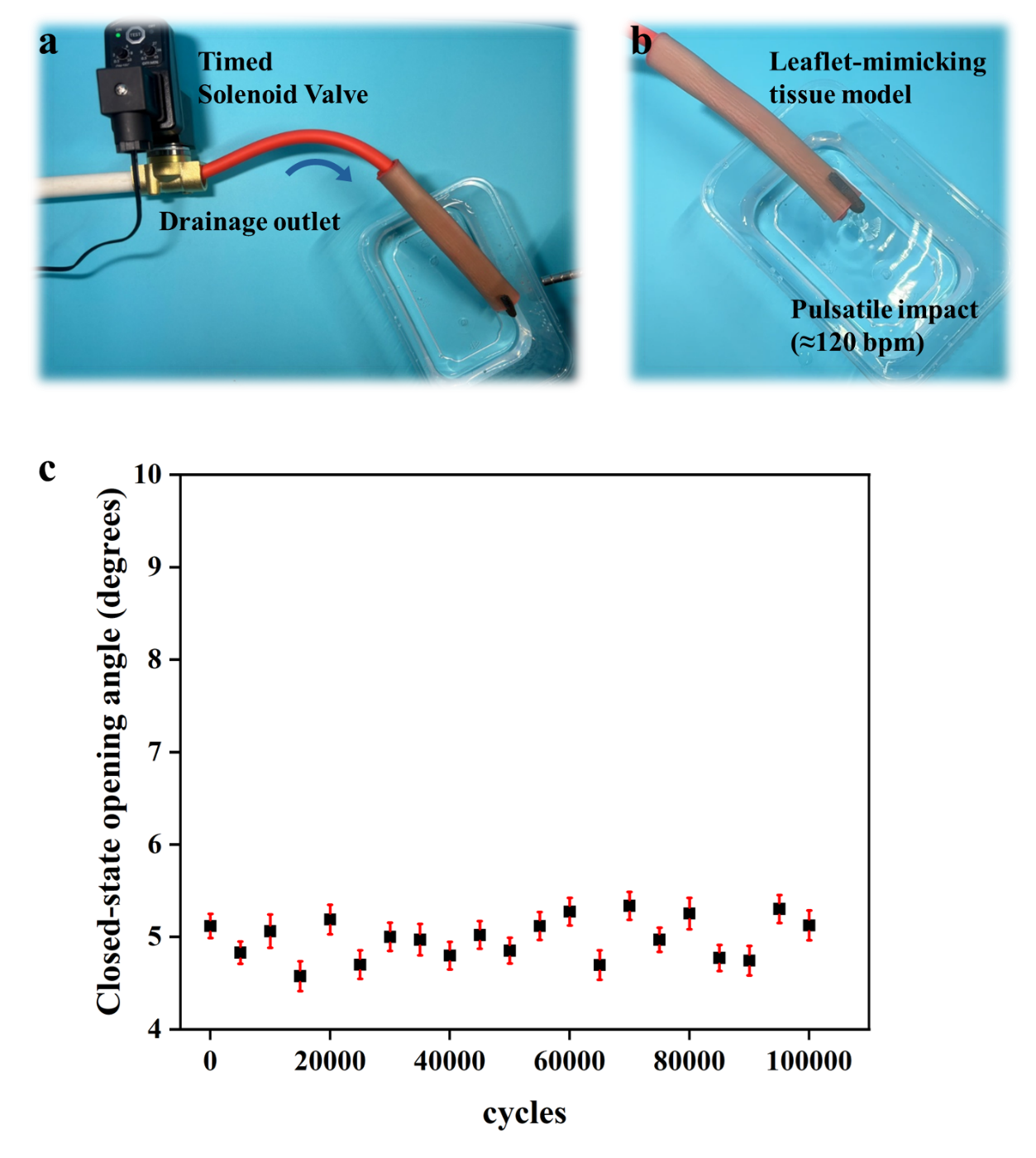


Figure S4. High-frequency cyclic pressure/flow impact test for dynamic clamping stability (a) Experimental setup of the high-pressure split-chamber solenoid-valve drainage system. The upper chamber was periodically and rapidly drained by a timed electromagnetic valve to generate pulsatile fluid impacts.(b) Close-up view of the leaflet-mimicking model with the pre-attached Y-shaped hydrogel clip during testing. The system delivered fluid impulses every 0.5 s (≈120 bpm) for 100,000 cycles to evaluate rhythmic hemodynamic loading–induced detachment or slippage. (c) Quantified closed-state angle drift during high-frequency cyclic impacts. The closed-state opening angle of the hydrogel clip was measured at predefined cycle intervals during rhythmic pressure (≈120 bpm) up to 100,000 cycles. Scatter points represent mean values and error bars indicate ±SD (n = 6). θ remained stable around 5° throughout cycling with no progressive increase or drift, confirming robust dynamic clamping stability under physiologically relevant pulsatile impacts.


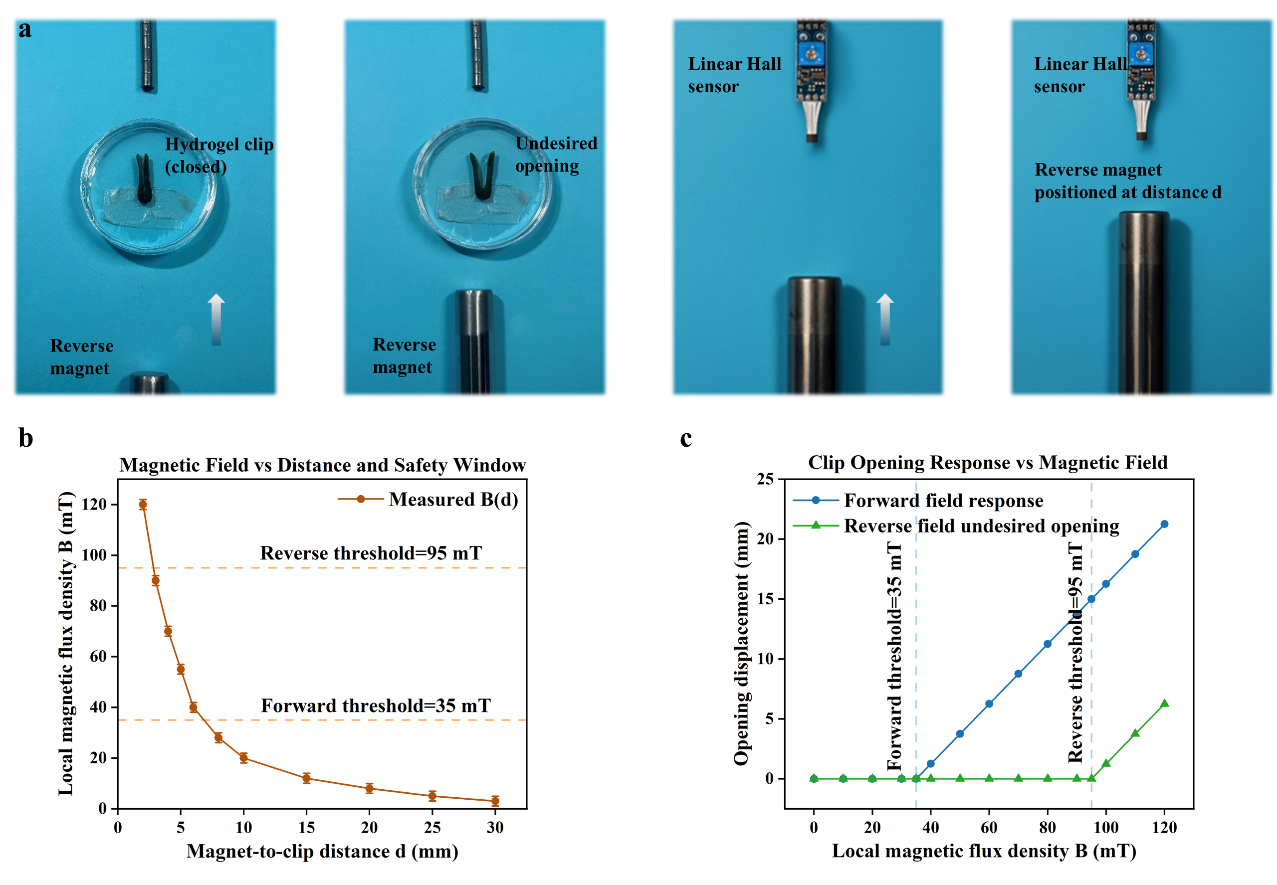


Figure S5. Reverse-field safety window and site-specific actuation characterization of the Y-shaped hydrogel clip. (a) Experimental setup for reverse-field mis-activation testing in hydrated PBS: the clip was first magnetically driven to a stable closed state, and an external magnetic field opposite to the normal actuation direction was then applied as a worst-case perturbation. A linear Hall sensor positioned adjacent to the clip continuously measured the local magnetic flux density $B$. (b) Spatial decay of the local magnetic flux density as a function of magnet–clip distance, calibrated by the Hall sensor. The forward actuation threshold for normal opening ($B_{\mathrm{th},\mathrm{open}}\approx35\text{ }\text{mT}$) and the reverse mis-activation threshold ($B_{\mathrm{th},\mathrm{reverse}}\approx95\text{ }\text{m}\text{T}$) are indicated, defining a quantifiable reverse-field safety window. (c) Opening-angle response of the clip versus local magnetic flux density. The clip shows reliable opening only above $B_{\mathrm{th},\mathrm{open}}$under forward fields, while remaining closed under reverse fields until $B_{\mathrm{th},\mathrm{reverse}}$is reached, demonstrating strong direction selectivity and site-specific activation.


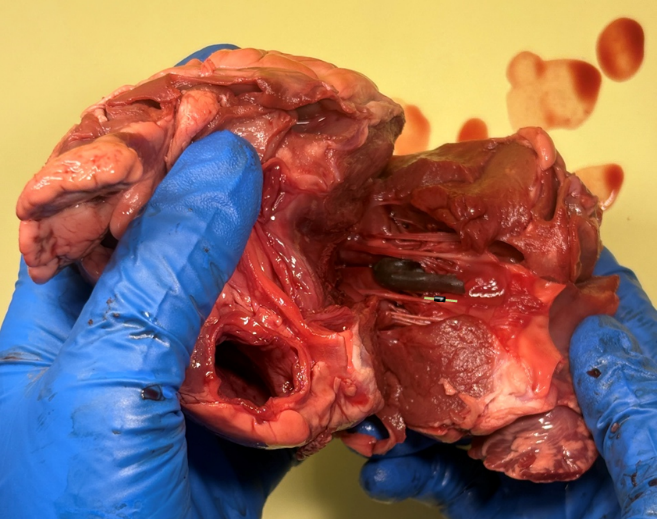


**Figure S6.** Ex vivo porcine mitral valve model for coaptation-length (CL) quantification after edge-to-edge clipping. Representative photograph of a freshly excised porcine heart showing the mitral valve apparatus after deployment of the Y-shaped hydrogel clip in an edge-to-edge configuration. This setup was used for objective measurement of leaflet coaptation length (CL): high-resolution images were captured from the valvular orifice direction with a calibrated scale, and CL was defined as the overlap length of the anterior and posterior leaflet free edges along the normal coaptation direction. CL was quantified in ImageJ by measuring three representative locations along the coaptation line and averaging them to obtain one CL value per sample ($n\geq5$). The revised Results include CL statistics, and achieving a predefined CL threshold—together with stable clamping force and absence of noticeable slippage—was adopted as a measurable criterion for successful leaflet approximation.
